# *Akkermansia muciniphila* and its membrane protein ameliorates intestinal inflammatory stress and promotes epithelial wound healing via CREBH and miR-143/145

**DOI:** 10.1186/s12929-023-00935-1

**Published:** 2023-06-07

**Authors:** Henry Wade, Kaichao Pan, Qihua Duan, Szczepan Kaluzny, Ekta Pandey, Linda Fatumoju, Viswanathan Saraswathi, Rongxue Wu, Edward N. Harris, Qiaozhu Su

**Affiliations:** 1 Institute for Global Food Security, School of Biological Sciences, Queen’s University Belfast, Belfast, BT9 5DL UK; 2 Department of Medicine, Section of Cardiology, University of Chicago, Chicago, USA; 3 Department of Biochemistry, University of Nebraska-Lincoln, Lincoln, NE 68583 USA; 4 University of Nebraska Medical Center, Omaha, NE 68198 USA

**Keywords:** *Akkermansia muciniphila*, Amuc_1100, CREBH, microRNA-143/145, Intestinal inflammatory stress, Epithelial regeneration, Wound healing

## Abstract

**Background:**

The intestinal epithelial barrier is the interface for interaction between gut microbiota and host metabolic systems. *Akkermansia muciniphila (A. muciniphila)* is a key player in the colonic microbiota that resides in the mucus layer, whose abundance is selectively decreased in the faecal microbiota of inflammatory bowel disease (IBD) patients. This study aims to investigate the regulatory mechanism among *A. muciniphila*, a transcription factor cAMP-responsive element-binding protein H (CREBH), and microRNA-143/145 (miR-143/145) in intestinal inflammatory stress, gut barrier integrity and epithelial regeneration.

**Methods:**

A novel mouse model with increased colonization of *A muciniphila* in the intestine of CREBH knockout mice, an epithelial wound healing assay and several molecular biological techniques were applied in this study. Results were analysed using a homoscedastic 2-tailed t-test.

**Results:**

Increased colonization of *A. muciniphila* in mouse gut enhanced expression of intestinal CREBH, which was associated with the mitigation of intestinal endoplasmic reticulum (ER) stress, gut barrier leakage and blood endotoxemia induced by dextran sulfate sodium (DSS). Genetic depletion of CREBH (CREBH-KO) significantly inhibited the expression of tight junction proteins that are associated with gut barrier integrity, including Claudin5 and Claudin8, but upregulated Claudin2, a tight junction protein that enhances gut permeability, resulting in intestinal hyperpermeability and inflammation. Upregulation of CREBH by *A. muciniphila* further coupled with miR-143/145 promoted intestinal epithelial cell (IEC) regeneration and wound repair via insulin-like growth factor (IGF) and IGFBP5 signalling. Moreover, the gene expressing an outer membrane protein of *A. muciniphila*, Amuc_1100, was cloned into a mammalian cell-expression vector and successfully expressed in porcine and human IECs. Expression of Amuc_1100 in IECs could recapitulate the health beneficial effect of *A. muciniphila* on the gut by activating CREBH, inhibiting ER stress and enhancing the expression of genes involved in gut barrier integrity and IEC’s regeneration.

**Conclusions:**

This study uncovers a novel mechanism that links *A. muciniphila* and its membrane protein with host CREBH, IGF signalling and miRNAs in mitigating intestinal inflammatory stress–gut barrier permeability and promoting intestinal wound healing. This novel finding may lend support to the development of therapeutic approaches for IBD by manipulating the interaction between host genes, gut bacteria and its bioactive components.

**Supplementary Information:**

The online version contains supplementary material available at 10.1186/s12929-023-00935-1.

## Background

The intestinal epithelium is the interface for the interaction between gut microbiota and host metabolic systems [1]. This barrier is enhanced by the presence of a mucus layer and immune factors produced by the host [2]. Accumulating evidence suggests that the development of inflammatory bowel disease (IBD) results from an inappropriate inflammatory response to intestinal microbes in a genetically susceptible host [3]. These observations have led to the conclusion that dysbiosis of the human gut microbiome is involved in either initiating or maintaining the disease [4, 5]. *Akkermansia muciniphila (A. muciniphila)* is one of the key players in the colonic mucus-associated microbiota that resides in the mucus layer of the intestine, a niche in close vicinity to host cells [6, 7]. *A. muciniphila* comprises a considerable population of the human gut microbiota, which contributes to about 1–4% of the faecal microbiota of healthy adults [8, 9], and is essential for mucus production in the human gut to maintain a healthy mucus layer and intestinal wall thickness [7, 10]. A decrease of this species has been found in human faeces and/or biopsies of several diseases, including IBD, appendicitis, type-2 diabetes and obesity [10, 11]. Increased intestinal colonization by *A. muciniphila* via direct administration has demonstrated the health beneficial effects of this gut bacteria on protecting mice from hyperlipidaemia, atherosclerosis, type-2 diabetes, and gut barrier disturbances [10–18]. Extracellular vesicles derived from this species are able to ameliorate colitis in mice, suggesting an important role of *A. muciniphila* in maintaining host intestinal homeostasis [19].

The cAMP-responsive element-binding protein H (CREBH, encoded by Creb3l3 in human) is a member of the CREB bZIP superfamily of transcription factors that is exclusively expressed in the liver and intestine. Hepatic CREBH was originally identified to be activated by ER stress and regulated the acute phase response gene expression [20]. Further studies revealed that CREBH is essential to hepatic glucose, lipid, and lipoprotein metabolism [21, 22]. CREBH has also been reported to inhibit inflammatory responses by suppressing nuclear factor (NF)-B (NFκB) signalling and expression of inflammatory chemokines which impeded the subsequent recruitment of immune cells (e.g., neutrophils) and inflammatory response [23, 24]. In addition, overexpression of CREBH in hepatic-specific CREBH transgenic mice supresses metabolic inflammation in white adipose tissue and improves high fat diet-induced obesity and insulin resistance [25], indicating the diverse role of CREBH in inflammatory responses. However, compared to hepatic CREBH, our knowledge on the pathophysiological function of intestinal CREBH is very limited. Clinically, a defective cAMP signalling pathway has been reported to be associated with the onset of paediatric colitis in children. Improvement of clinical symptoms after treatment was associated with the restoration of cAMP signalling [26]. Although CREBH is a key molecule in the cAMP pathway, its role in the intestinal inflammatory disease is unknown. In situ hybridization analysis revealed that CREBH mRNA is present in the intestinal epithelial cells (IECs) of the villi but not the crypts [27], which may suggest a potential interaction between CREBH and the mucosal resident gut bacteria.

The interaction between commensal bacteria and the host toll-like receptors (TLR2 and TLR4) under physiological conditions plays a crucial role in the maintenance of intestinal epithelial homeostasis by modulating the expression of various tight-junction proteins [28]. The epithelial tight junction proteins (e.g., claudin5 [CLDN5] and claudin8 [CLDN8]) which seal the paracellular space between IECs are crucial in maintaining the gut barrier integrity by controlling the permeability of essential ions, nutrients, and water but restricting the leakage of bacterial toxins and pathogens that may cause intestinal inflammation [29, 30]. The tight junction is composed of several transmembrane and cytosolic proteins, including occludin (OCLN), claudins (CLDNs), zonula occludens (ZOs), tricellulin, cingulin, and junctional adhesion molecules (JAM) which interact with each other and with the cytoskeleton to form a complex architecture [31]. Together with intracellular signalling proteins, tight junction complexes maintain gut barrier integrity and regulate paracellular permeability [32]. Alterations in CLDN levels affect the intestinal barrier permeability in different ways depending on the type of CLDN isoform [33]. For example, downregulation of CLDN5 and/or CLDN8 drastically reduces barrier integrity. In contrast, upregulation of claudin2 (CLDN2), a leaky gut barrier mediator promotes inflammation and IBD [31, 34, 35]. This evidence highlights the diverse functions of tight junctions in gut barrier integrity, and therefore the regulatory mechanisms, which are not fully clear.

The re-establishment of the epithelial barrier after mucosal injury in IBD depends on a continuous supply of epithelial cells under basal conditions and is regulated by growth hormones [36–38]. The insulin-like growth factor (IGF) signalling pathway functions in mediating cell proliferation, apoptosis, differentiation, survival, metabolism, and migration. IGF1 is mainly secreted by the liver and binds to the IGF1 receptor (IGF1R) with high affinity and to the IGF2 receptor (IGF2R) with lower affinity. The gastrointestinal (GI) tract is a major target organ of IGF action that stimulates IEC proliferation and promotes recovery of intestinal injury from a variety of pathological conditions, including radiation injury and dextran sulfate sodium (DSS)-induced colitis [39]. Intestinal smooth muscle cells are also abundant sources of IGF1 that act in an autocrine and paracrine fashion to regulate cell growth, survival and response to inflammation [40]. In pathological conditions, e.g. Crohn’s disease (CD) and malnutrition, IGF1 expression and secretion are markedly reduced [40]. The IGF binding proteins (IGFBPs), designated IGFBP 1–6, typically function to competitively inhibit IGF signalling. For instance, IGFBP5 negatively regulates IGF signalling by binding and sequestering IGF ligands, resulting in inhibition of epithelial IGF1 signalling and subsequent blunting of the epithelial repair system [41]. In addition, the cAMP responsive element binding protein (CREB) has also been reported to positively regulate IGF1 signalling through interaction with hepatic nuclear factor 1 alpha (HNF1α) in IGF1 gene promoter and enhanced IGF1 expression in the liver of a grass carp model [42], implicating the involvement of CREB family in the IEC regeneration. However, whether intestinal CREBH is involved in mammalian IEC proliferation and how it interacts with gut microbiome in maintaining epithelial homeostasis remain unclear.

The present study demonstrated that increased colonization of *A. muciniphila* in the colon of mice upregulated and activated intestinal CREBH, which subsequently mitigated intestinal endoplasmic reticulum (ER) stress, gut barrier leakage and blood endotoxemia induced by DSS. The mechanistic study revealed that CREBH positively regulated the expression of tight junctions that contribute to gut barrier integrity (e.g., CLDN5 and CLDN8) but inhibited a leaky gut tight junction CLDN2, leading to the inhibition of gut permeability and inflammation. Upregulation of CREBH by *A. muciniphila* further enhanced the expression of microRNA-143/145 (miR-143/145) to target IGFBP5, which improve IGF-mediated IEC regeneration and epithelial cell wound repair. Moreover, expression of an *A. muciniphila* outer membrane protein, Amuc_1100, in porcine small intestinal epithelial cell line (IPEC-J2) and human Caco2 cells enhanced the expression and activation of CREBH, which further induced expression of genes involved in gut barrier function and epithelial regeneration. The TLR2 and TNF receptor associated factor 6 (TRAF6) were found to mediate activation of CREBH by Amuc_1100. This study uncovers a novel regulatory circuit linking *A. muciniphila* and its membrane protein Amuc_1100 with host CREBH, IGF signalling, and microRNAs in mitigating intestinal epithelial ER stress and gut barrier permeability as well as promoting intestinal epithelial regeneration.

## Methods

### Cell culture, transfection, and treatments

Normal porcine small intestinal epithelial cell line (IPEC-J2) and human colonic adenocarcinoma (Caco2) cells were obtained from America Type Culture Collection (ATCC, Manassas, VI, USA) and maintained in Gibco DMEM/F12 (1:1) (11330032) containing 10% FBS and 1% penicillin/streptomycin (Gibco, 15140-122) or Gibco MEM (11,583,397) containing 10% FBS, 1% l-Glutamine (Gibco, 25030-081), and 1% penicillin/streptomycin (Gibco, 15140-122), respectively. Cells were grown in T75 flasks at 37 °C, 5% CO_2_. All cell treatments were conducted in standard 6-well plates with 3 × 10^5^ cells seeded per well and maintained in the described media. For Tumour necrosis factor (TNF)α treatment, when cells reached 60–70% confluence, cells were treated with 20 ng/mL recombinant human TNFα (Gibco: PHC3015) for a time course of 12 or 24 h. Interleukin (IL)-1β treatment was performed under identical conditions and treated with 10 ng/mL human IL-1β (Gibco, PHC0814) for 24 or 48 h. For transfection, 3 × 10^5^ cells were seeded in wells of 6-well plates and grown to 60–70% confluence in described media. 1 h prior to transfection, media was replaced with serum and antibiotic free Gibco MEM (11,583,397) containing 1% l-Glutamine. Cells were then transfected with 2 µg of plasmid: either empty pFLAG-CMV-2 vector, pFLAG-CMV-2-CREBH (N-terminal CREBH, constitutively active form), empty pcDNA3.1-Flag vector, or pcDNA3.1-Flag-Amuc_1100, respectively. Transfections were carried out using Lipofectamine 3000 (Invitrogen, L3000-008) with Opti-MEM media (Gibco, 31985-047) for 6 h, then media was replaced with standard growth media and cells were allowed to grow for additional 48 h. Total RNA or protein lysates were extracted at the end of each treatment.

### Animal protocols

All animal experiments were approved by the University of Nebraska-Lincoln Institutional Animal Care and Use Committee (Protocol #2414) and were carried out under the institutional guidelines for ethical animal use. Wild type (WT) (C57BL/6J) and CREBH-KO mice with exons 4–7 of the CrebH gene deleted as previously described [20] at 12–18 weeks of age were used in this study (n = 5–10/per group). Animals were housed with alternating 12 h light and dark cycles with free access to food and water and placed on a chow diet (Dyets Inc., USA). For DSS treatment, both WT and CREBH-KO mice were divided into two groups and supplied with drinking water containing 1.5% DSS or vehicle (Veh, H_2_O) with free access to food for 14 days to induce IBD. Body weights and food intakes were monitor every other days. Plasmas, ileum, and colon tissues were harvested at the end of treatment for further analysis. For TNFα treatment, both WT and CREBH-KO mice were divided into two groups followed by fasting for 6 h with free access to water. Mice were then treated with either recombinant mouse TNFα (60 ng/g body weight, IP injection) or saline control. Plasma, ileum and colon tissues were collected at 5 h post-treatment under anesthetization with isoflurane (3% mixed with oxygen). Intestinal tissues were then homogenized in solubilization buffer to prepare protein lysates or subjected to RNA extraction for further analysis.

### *A. muciniphila* and IBD treatment

WT and CREBH-KO mice were pre-treated with either 200 µL 1 × 10^9^ CFU/mL live *A. muciniphila* suspended in 2.5% glycerol/PBS or 200 µL PBS containing 2.5% glycerol as control every other day for 6 days via oral gavage. At the end of *A. muciniphila* treatment, the mice were supplied with drinking water containing 1.5% DSS for 14 days to induce IBD. Plasma, ileum, and colon tissues were harvested at the end of treatment for further analysis.

### FITC-dextran (FD4) permeability assay

In vivo intestinal permeability assay to determine barrier function was performed using an Fluorescein Isothiocyanate (FITC)-labelled dextran method as described [43]. Briefly, mice were orally gavaged with permeability tracer (0.4 mg/g body weight of FITC-labelled dextran, MW 4000; Sigma-Aldrich) after fasting for 12 h. Serum was collected retro-orbitally after 4 h. Fluorescence intensity of each sample was measured (excitation, 492 nm; emission, 525 nm, SYNERGY H1 microplate reader), and FITC-dextran concentrations were determined from standard curves generated by serial dilution of FITC dextran.

### Construction of Amuc_1100 expression plasmid

*A. muciniphila* genomic DNA was isolated using Trizol (Life Technologies, Grand Island, NY, USA) following manufacturer protocol for DNA extraction. PCR amplification of Amuc_1100 cDNA reading frame from *A. muciniphila* genomic DNA was performed using the following primers: Amuc_1100-Fwd: 5′-ATAAGCTTATGAGCAATTGGATTACAGACAACAAGCCCG-3′, Amuc_1100-Rev: 5′-TATGGGCCCTTAATCTTCAGACGGTTCCTGAGCCTTGGG-3′. Amuc_1100 was purified using QIAquick^®^ Gel Extraction Kit (28704) following the manufacturer protocol. The cDNA of Amuc_1100 reading frame was cloned into the pcDNA3.1-Flag expression vector by restriction digestion with Hind III (Promega: R604A) and Apa I (Promega: R636A) at 37 °C for 1 h followed by ligation overnight at room temperature. The construct was verified by sequencing the entire cDNA open reading frame of the protein.

### RNA isolation, reverse transcription and qRT-PCR

Total RNA was isolated from tissue using TRIzol (Life Technologies, Grand Island, NY, USA). RNA integrity was confirmed using a NanoDrop 2000 (Wilmington, DE). First strand cDNA was synthesized with oligo (dT) and random primers using a High-Capacity cDNA Reverse Transcription Kit with RNase Inhibitor (Life Technologies). Quantification of gene expression was performed by SYBR Green qPCR on a Roche LightCycler 480 Instrument II. Relative expression of gene mRNA was calculated using the expression of 18s rRNA for normalization. Sequences of the primers used in this study are listed in Additional file 1: Table S1.

### Immunoblot analyses

 Immunoblotting analysis was performed as previously described [44]. The following primary antibodies were used: anti-Claudin-2 (Life Technologies; 325,600), anti-Claudin-3 (Thermo Scientific; PA5-16867), anti-Occludin (Invitrogen; 40-4700), Anti-CREB3L3 (Santa Cruz Biotechnology; SC-69,375), anti-IGFBP5 (Cell Signalling; 10,941S), anti-IGF-1 (Abcam; ab9572), anti-SAPK/JNK (Cell Signalling; 9252T), anti-p-SAPK/JNK (Cell Signalling; 9255S), anti-p-EIF2α (Cell Signalling, 3597S). Secondary antibodies utilised: anti-Mouse (Cell signalling; 7076S), anti-Rabbit (Cell Signalling, 7074S), anti-Goat (Abcam; 7076S). All antibodies were used at a final concentration of 0.1–1 µg/mL. After incubation with the appropriate horseradish peroxidase-conjugated anti-mouse (GE Healthcare: NA931V) and anti-rabbit (GE Healthcare: NA934V) IgG secondary antibodies (1:2000 dilution), signals were detected using enhanced chemiluminescence (Pierce, Rockford IL, USA).

### Plasma endotoxin (LPS) measurement

Plasma endotoxin (LPS) concentrations were determined using the Pierce LAL Chromogenic Endotoxin Quantitation Kit (#88282, Thermo Scientific, Rockford IL, USA) according to manufacturer’s protocol. Briefly, plasma was diluted 50-fold and heat shocked at 70 ºC for 15 min and then stored at -20ºC until use. The samples were incubated with LAL reagent at 37 ºC for 10 min in sterile 96 well plates, followed by incubation with chromogenic substrate supplied in the kit. The reaction was stopped using 25% acetic acid (Stop Reagent) and absorbance was measured at 405 nm using a Tecan Safire microplate reader. The concentration of endotoxin in plasma was determined using a standard curve prepared using Endotoxin Standard solutions.

### Statistical analyses

The Anderson-Darling test on the residuals was used to test normality and Levene’s test was used to test for equal variances. The two-tailed Student’s t-test was used for statistical analyses of two-group comparisons. All results are presented as means ± SD. Asterisks (* or **) indicate statistically significant differences of p < 0.05 or p < 0.01, respectively, compared to controls.

## Results

### *A. muciniphila* ameliorates intestinal inflammatory and ER stress and endotoxemia in DSS-induced colitis

**Fig. 1:**
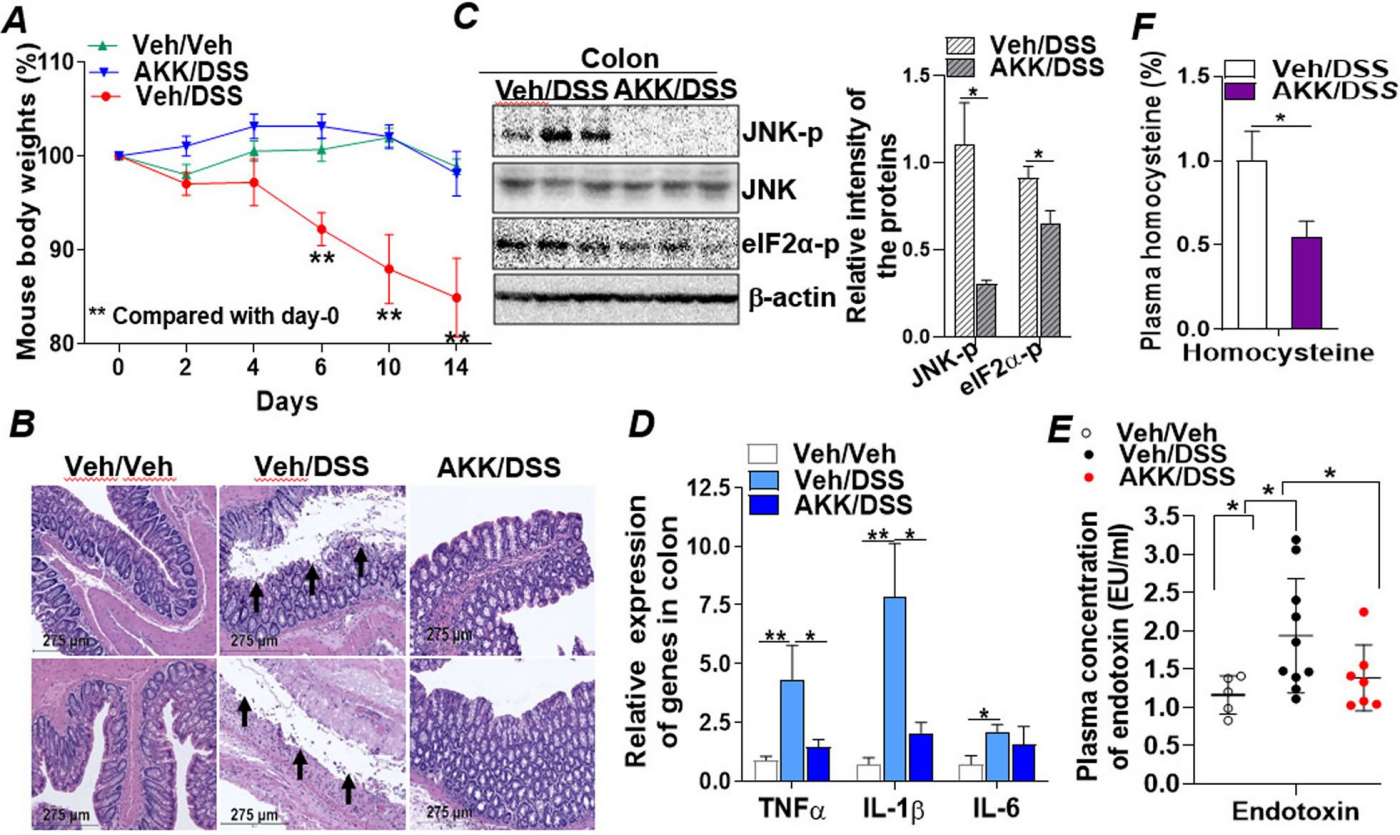
*A. muciniphila* ameliorates intestinal inflammatory and ER stress and endotoxemia in DSS-induced colitis. The indicated groups of wild type (WT, C57BL/6J) mice (n = 5–10/groups) were pre-treated with *A. muciniphila* (AKK) or Veh (PBS) followed by DSS or Veh (H_2_O) treatment as described in the "Methods". Plasmas and colon tissues were collected for protein and RNA extractions for the following analysis. **A** Mouse body weight (%) at day-0 to day-14. **B** Representative histological images of distal colon tissues by H&E staining as described in the Additional file 1: Supplemental Methods. Scale bars, 275 μm. **C** Immunoblotting analysis of ER-stress markers phospho-JNK, JNK, phospho-eIF2α, and loading control β-actin in the colon tissues of Veh/DSS or AKK/DSS treated mice. **D** mRNA expression of inflammatory cytokines TNFα, IL1β, and IL-6 in the colons of Veh/Veh, Veh/DSS or AKK/DSS treated mice. **E** Plasma endotoxin (LPS) concentrations from the Veh (PBS)/Veh (H_2_O), Veh/DSS or AKK/DSS treated WT mice. **F** Homocysteine in the plasmas of Veh/DSS or AKK/DSS treated WT mice determined by metabolomics analysis. Results represent the means ± SD. The two-tailed Student’s t-test was used for statistical analyses of two-group comparisons. *P < 0.05 and **P < 0.01 versus controls

The abundance of *A. muciniphila* (AKK) has been reported to be significantly decreased in patients with IBD and in mice with colitis. However, the underlying molecular mechanism remains unclear. We and other researchers previously reported that administration of live *A. muciniphila* via oral gavage could successfully increase colonization of *A. muciniphila* in the mouse intestine [44]. To investigate the mechanism for increased colonic colonization of *A. muciniphila* improving gut inflammation, three groups of C57/B6 mice were pre-treated with a dose of either *A. muciniphila* (200 µL, 1 × 10^9^ cfu/mL) in PBS with 2.5% glycerol, or the vehicle (PBS with 2.5% glycerol) as control through oral gavage every other day for 6 days. Next, two groups of these mice were further supplied with drinking water containing DSS to induce colitis while the third group was supplied with regular drinking water to serve as negative control as detailed in the "Methods". Treatment with DSS induced significant body weight decrease in the vehicle (PBS) pre-treated mice (Veh/DSS), starting at day-6 post-treatment compared to day-0, which was further decreased until the termination of the experiment at day-14 (Fig. 1A). In contrast, DSS failed to induce body weight loss in the *A. muciniphila* pre-treated mice (AKK/DSS), in which the mice maintained their body weights comparable to the negative control group (Veh/Veh) during the experimental time course (Fig. 1A). Histological analysis of mouse distal colon tissues by hematoxylin and eosin (H&E) staining revealed that pre-treatment with *A. muciniphila* attenuated colonic injuries induced by DSS treatment whereas, loss of intestinal histological structure and disruption of the epithelial barrier were observed in the vehicle treated group (Veh/DSS) upon DSS treatment (Fig. 1B). Clinical severity of the colitis was further evaluated using a disease activity index (DAI) that incorporates body weight loss, stool consistency, and GI tract bleeding as detailed in Additional file 1: Table S2 [45], which showed that treatment with *A. muciniphila* significantly improved DAI (Additional file 1: Fig. S1A), implicating the protective effect of *A. muciniphila* on mitigating the development of colitis associated pathological symptoms. Immunoblotting analysis of ER stress markers in the colons revealed that *A. muciniphila* treatment inhibited activation of ER stress markers, phospho-c-Jun N-terminal kinase (JNK-p) and phospho-eukaryotic translation initiation factor 2α (eIF2α-p) (Fig. 1C). q-RT-PCR analysis of inflammatory cytokine gene expression also showed a significant induction of TNFα, IL-1β and IL-6 genes in the vehicle treated mice, which was inhibited in the *A. muciniphila*-treated mice (Fig. 1D). Immunohistochemistry (IHC) analysis with an anti-F4/80 antibody further demonstrated that *A. muciniphila* alleviated infiltration of macrophages in the colonic tissues upon DSS treatment (Additional file 1: Fig. S1B). Together, these data demonstrated an improvement of intestinal inflammatory and ER stress in the colons of AKK/DSS treated mice compared to the Veh/DSS treated mice.

*A. muciniphila* is a Gram-negative anaerobic gut bacterium that contains lipopolysaccharides (LPS) in its outer cell wall. It has been reported that circulating levels of LPS in host blood depend on the release of LPS from gut bacteria and is associated with gut permeability [46]. IBD compromises gut barrier integrity, particularly intestinal epithelial tight junction function, which increases gut permeability and induces metabolic endotoxemia (high level of LPS in the blood circulation) [46]. To determine whether increased *A. muciniphila* in the GI tract will elevate host plasma LPS levels, an endotoxin (LPS) assay was conducted to determine plasma LPS concentration. As shown in Fig. 1E, plasma endotoxin (LPS) concentrations were significantly elevated in the Veh/DSS-treated mice compared to the control group (Veh/Veh) (Fig. 1E). Pre-administration of *A. muciniphila* in the DSS-treated mice (AKK/DSS) reduced plasma LPS to a level similar to the control (Fig. 1E). Next, a metabolomics analysis of mouse plasma was conducted to further determine the impact of *A. muciniphila* on systemic metabolic inflammation. Consistent with the improvement of local inflammation in the gut, biomarker of metabolic inflammation, homocysteine, was markedly reduced in the AKK/DSS mice compared to the Veh/DSS group (Fig. 1F). Together, these data demonstrate the anti-inflammatory effect of *A. muciniphila* in mitigating intestinal epithelial inflammatory stress and systemic metabolic inflammation induced by DSS.

### Upregulation of CREBH by *A. muciniphila* promotes intestinal barrier integrity

**Fig. 2:**
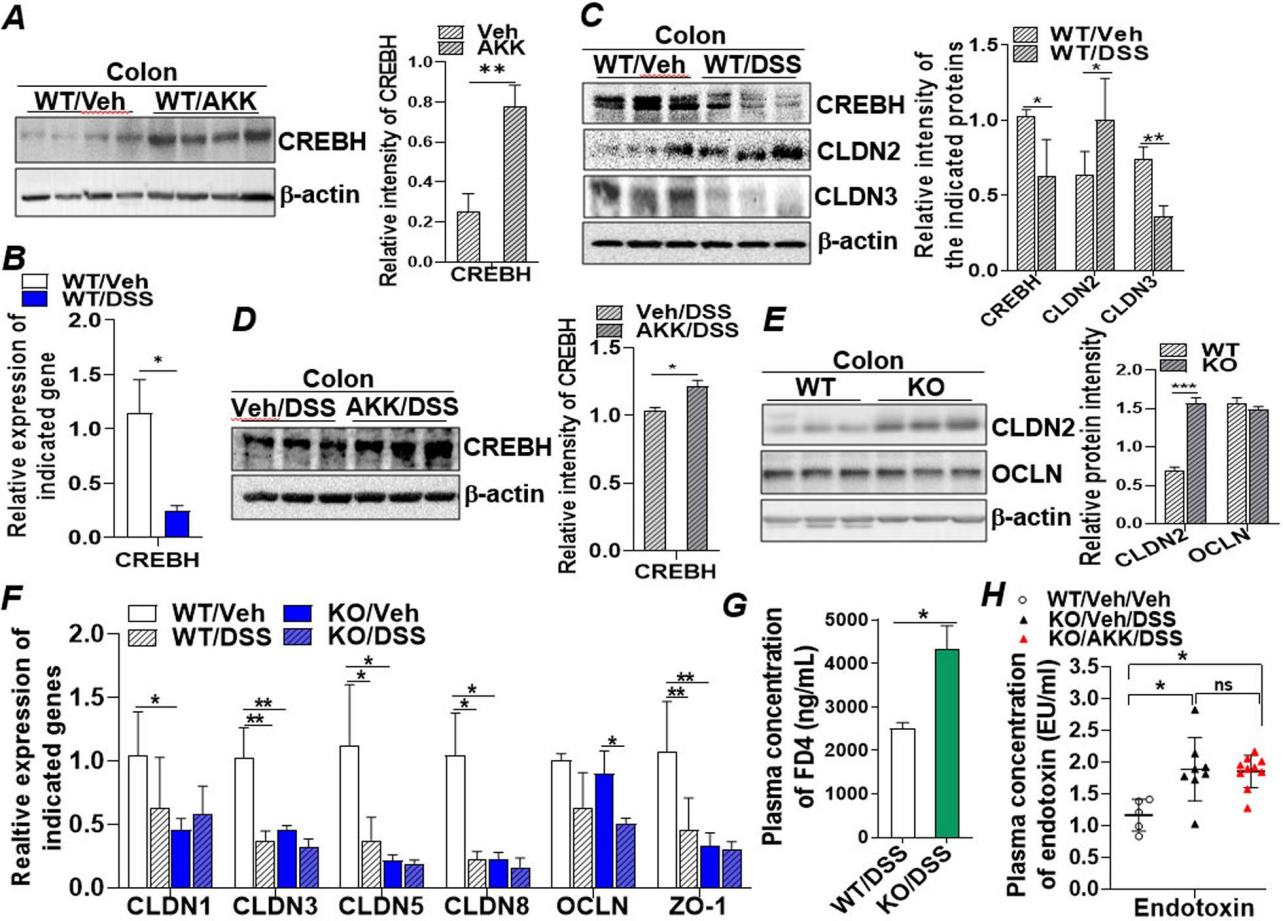
Upregulation of CREBH by *A. muciniphila* promotes intestinal barrier integrity. **A** Immunoblotting analysis of CREBH and loading control β-actin proteins in the colon tissues of WT mice treated with a dose of AKK or Veh every other day for 14 days. **B** mRNA expression of CREBH in the Veh or DSS treated colons determined by qRT-PCR. **C** Immunoblotting analysis of CREBH, CLDN2, CLDN3 and β-actin in the colons of mice treated with Veh or DSS. **D** Immunoblotting analysis of CREBH and β-actin in the colon tissues of the mice pre-treated with Veh or AKK followed by Veh or DSS treatment. **E** Immunoblotting analysis of CLDN2, OCLN and β-actin in the colon tissues of the WT and CREBH-KO mice. **F** qRT-PCR analysis of mRNAs of CLDN1, 3, 5, and 8, OCLN and ZO-1 in the colon tissues of WT or CREBH-KO mice treated with Veh or DSS. **G** FD4 gut permeability assay: two groups of DSS-treated WT and KO mice were subjected to a FD4 permeability assay as described in the "Methods". Plasma FD4 concentrations were determined and presented as means ± SD. **H** Plasma endotoxin (LPS) concentrations from the control WT/Veh/Veh, KO/Veh/DSS or KO/AKK/DSS treated mice determined as described in the "Methods". Results represent the means ± SD. The two-tailed Student’s t-test was used for statistical analyses of two-group comparisons. *P < 0.05 and **P < 0.01 versus controls

To investigate the anti-inflammatory mechanism of *A. muciniphila* in the intestinal inflammation, we focused on CREBH, a transcription factor exclusively expressed in the liver and intestine and involved in inflammatory and cAMP signalling. Moreover, intestinal CREBH is expressed in the epithelial cells of the villi [27], which may facilitate its crosstalk with gut bacteria in the mucus layer. Immunoblotting analysis revealed significant induction of CREBH protein in the colon of *A. muciniphila* treated mice (Fig. 2A). DSS treatment suppressed mRNA and protein expressions of colonic CREBH (Fig. 2B, C), which was accompanied with increased expression of a leaky gut barrier mediator CLDN2, but reduced expression of protective tight junction protein, CLDN3 (Fig. 2C). Pre-treatment with *A. muciniphila* rescued CREBH from the inhibition by DSS at both mRNA and protein levels (Fig. 2D; Additional file 1: Fig. S2A). Further investigating the regulation of CREBH on intestinal claudins in the CREBH knockout (CREBH-KO) mice revealed that genetic depletion of CREBH significantly upregulated leaky gut mediator, CLDN2, but downregulated mRNA levels of other tight junction proteins that maintain gut barrier integrity, including CLDN1, CLDN3, CLDN5, CLDN8 and ZO-1 but not OCLN (Fig. 2E, F). DSS treatment inhibited expression of CLDN1, CLDN3, CLDN5, CLDN8 and ZO-1 in the WT mice to a similar level of CREBH-KO (Fig. 2F). The essential role of CREBH on intestinal barrier integrity was further determined by a FITC-labelled dextran assay, which demonstrated CREBH-KO enhanced DSS-induced gut permeability, allowing more FD4 molecules to permeate through the gut barrier and get into blood circulation, which resulted in notably higher concentration of FD4 in the plasmas of DSS-treated CREBH-KO mice (Fig. 2G). Furthermore, depletion of CREBH abrogated the protective effect of *A. muciniphila* on DSS-induced body weight loss and endotoxemia in mice (Fig. 2H; Additional file 1: Fig. S2B). Together, these data demonstrated that CREBH is crucial in maintaining intestinal barrier integrity by positively upregulating expression of the protective tight junction proteins. Depletion of CREBH predisposes mice to DSS-induced colitis, and an intact CREBH is essential to mediate the gut health beneficial effect of *A. muciniphila.*

### CREBH enhances the expression of tight junction proteins that were impaired by inflammatory cytokines

**Fig. 3:**
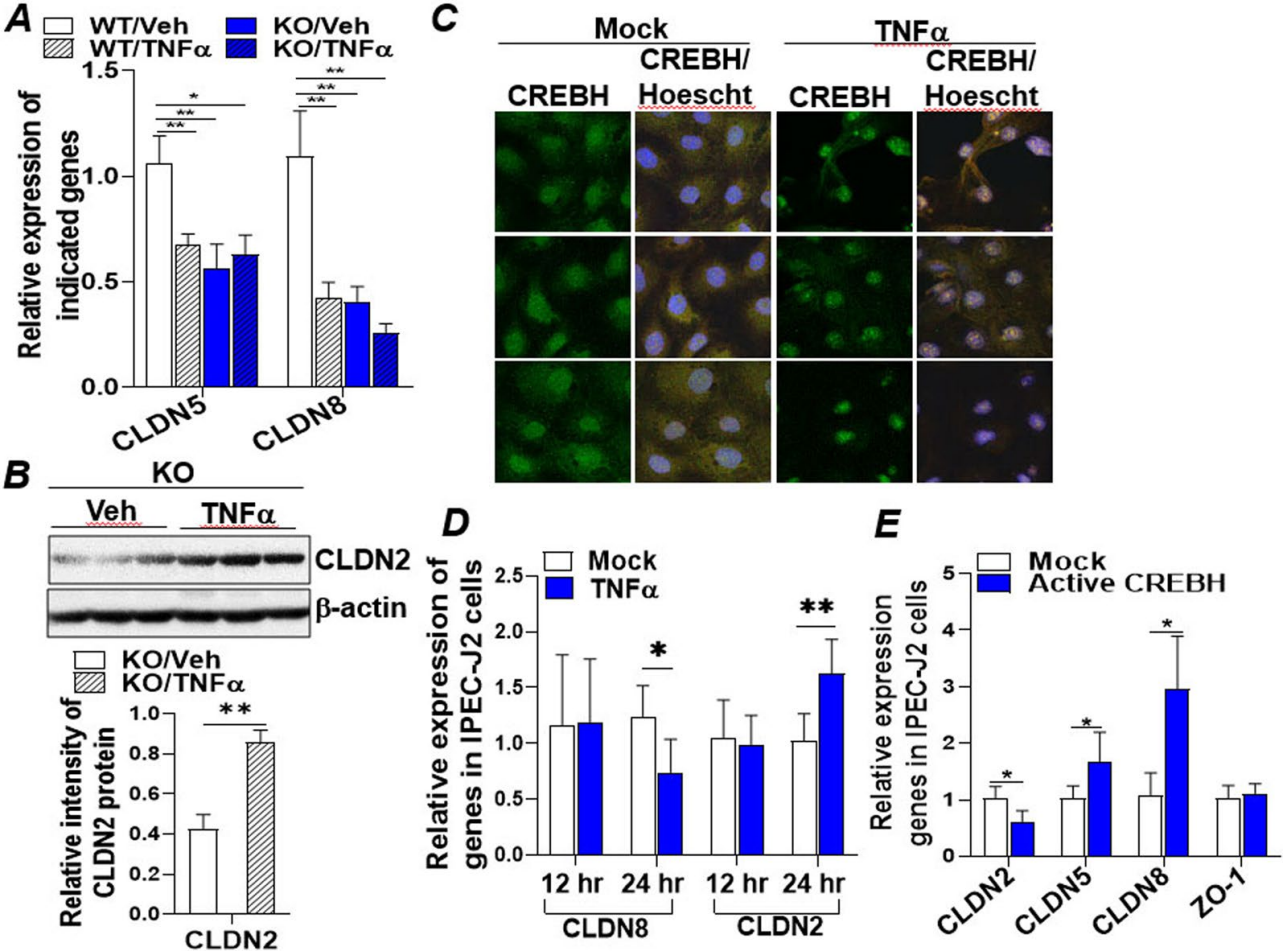
CREBH enhances the expression of intestinal integrity associated tight junction proteins impaired by inflammatory cytokines. Four groups of WT or CREBH-KO mice (n = 5–6/group) were treated with either TNFα (60 ng/g body weight) or vehicle (Veh, saline) for 5 h. Ileum tissues were collected for protein and RNA extraction for the following analyses: **A** mRNA expression of CLDN5 and CLDN8 in the ileums measured by qRT-PCR. **B** Immunoblotting analysis of CLDN2 and β-actin in the ileums of the KO mice treated with TNFα or Veh. **C** Immunofluorescent confocal microscopic images of IPEC-J2 cells treated with Veh (H_2_O) or TNFα (20 ng/mL) for 24 h followed by stained with anti-CREBH antibody or Hoescht 33,342 as described in the Additional file 1: Supplemental Methods. **D** qRT-PCR analysis of mRNA expression of CLDN8 and CLDN2 in IPEC-J2 cells treated with Veh (H_2_O) or TNFα (20 ng/mL) for 12 or 24 h. **E** IPEC-J2 cells were transfected with mock (empty vector) or an active CREBH (N-terminal CREBH, constitutively active) plasmid for 48 h. Total RNAs were extracted for measurement of mRNA expression of CLDN2, 5, 8, and ZO-1 by qRT-PCR. Results represent the means ± SD. The two-tailed Student’s t-test was used for statistical analyses of two-group comparisons. *P < 0.05 and **P < 0.01 versus controls

The protective effect of CREBH on gut integrity was further investigated in a more physiologically relevant mouse model with intestinal inflammation. Four groups of WT and CREBH-KO mice were treated with either control vehicle (saline) or inflammatory cytokine TNFα for 5 h to induce intestinal inflammation. q-RT-PCR analysis revealed that TNFα treatment significantly inhibited mRNA expression of CLDN5 and CLDN8 in the ileum (Fig. 3A). Depletion of CREBH suppressed expression of CLDN5 and CLDN8 mRNAs to a level similar to that of WT mice treated with TNFα (Fig. 3A). Immunoblotting analysis further showed that TNFα upregulated expression of the leaky gut mediator CLDN2 protein in the ileum of CREBH-KO mice (Fig. 3B). In vitro, treatment of a porcine intestinal epithelial cell line (IPEC-J2) with TNFα (20 ng/mL) for 12 and 24 h inhibited expression of CREBH which was accompanied by elevated CLDN2 and reduced CLDN8 mRNA at 24 h post-treatment (Fig. 3C, D). Upregulation of CLDN2 by TNFα was further confirmed in a human colonic adenocarcinoma cell line (Caco2) treated with TNFα (20 ng/mL) or IL-1β (10 ng/mL), respectively (Additional file 1: Figs. S3A, B). Next, a gain-of-function assay was conducted to determine the direct regulation of CREBH on tight junction proteins. We transfected IPEC-J2 cells with a constitutively active form of CREBH (N-terminal CREBH) or an empty vector (Mock) and found that overexpression of CREBH significantly inhibited mRNA of CLDN2 but enhanced expression of CLDN5 and CLDN8 (Fig. 3E). Similar changes were observed in CREBH-transfected Caco2 cells in which mRNAs of CLDN5, CLDN8 and OCLN but not ZO-1 were upregulated upon expression of CREBH (Additional file 1: Fig. S3C). Interestingly, treating the CREBH-transfected Caco2 cells with TNFα reversed the increased CLDN5 and OCLN mRNAs back to a level similar to the mock control (Additional file 1: Fig. S3D). Together, these data demonstrated that CREBH enhances the expression of tight junction proeins that are disturbed by inflammatory cytokines to sustain gut barrier integrity in vivo and in vitro.

### CREBH promotes intestinal epithelial regeneration and wound healing via IGF signalling

**Fig. 4:**
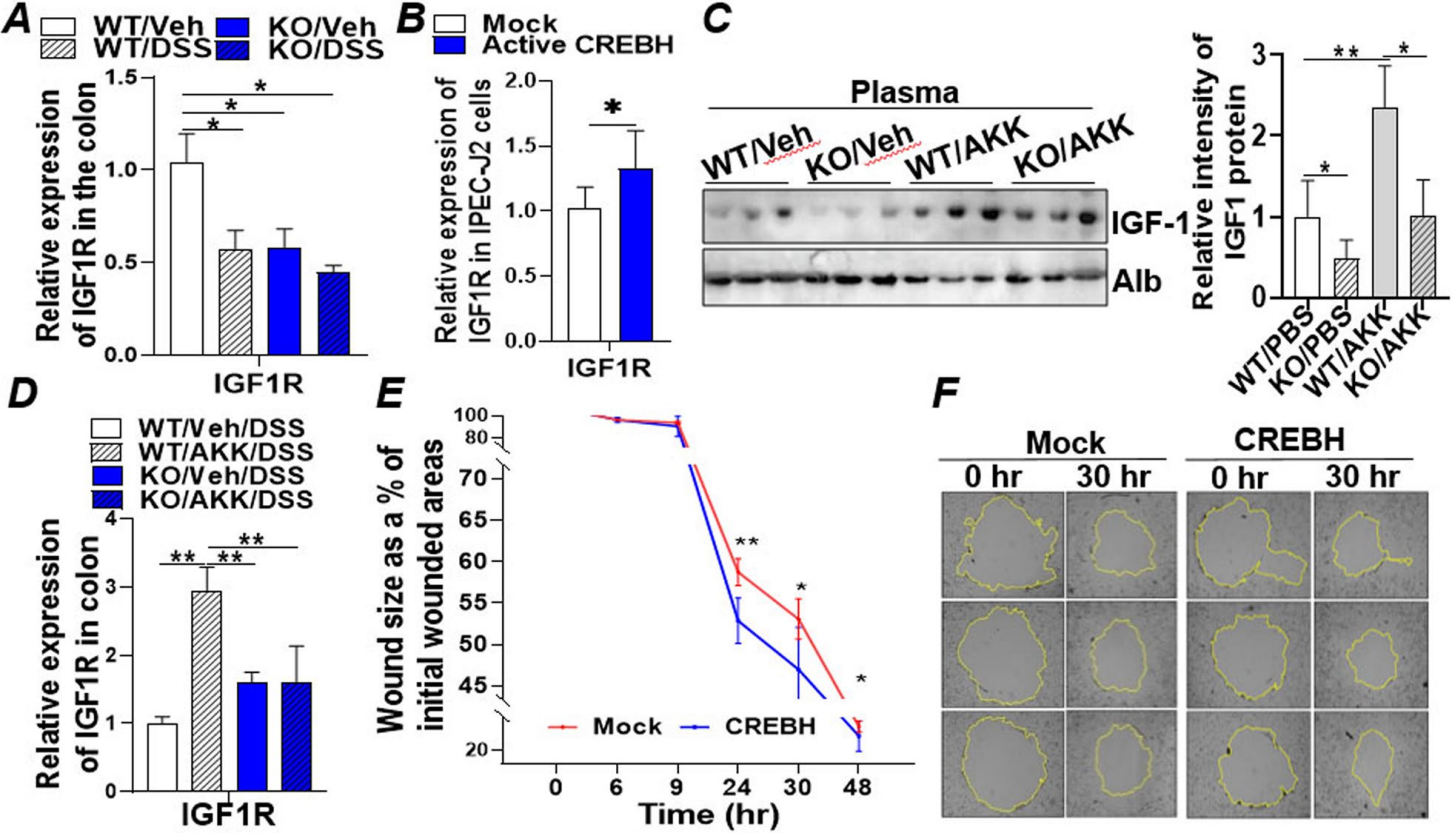
CREBH promotes intestinal epithelial regeneration and wound healing via mediation of IGF signalling. **A** mRNA expression of IGF1R in the colon tissues of wild type (WT, C57BL/6J) and CREBH-KO mice (n = 5–10/groups) treated with DSS or vehicle (Veh, H_2_O) in drinking water for 14 days. **B** qRT-PCR analysis of mRNA expression of IGF1R in IPEC-J2 cells transfected with mock (empty vector) or an active CREBH (N-terminal CREBH, constitutively active) plasmid for 48 h. **C** Immunoblotting analysis of plasma IGF-1 and loading control Albumin in the plasmas of WT and CREBH-KO mice (n = 5–6/group). **D** mRNA expression of IGF1R in the colon tissues of WT and CREBH-KO mice (n = 5–10/groups) pre-treatment with *A. muciniphila* (AKK) or Veh (PBS) followed by DSS or Veh treatment as described in the "Methods". **E** IEC wound healing assay: Caco2 cells transfected with mock (empty vector) or an CREBH expressing plasmid were grown to a confluent monolayer before being wounded by scratching and monitored for rate of wound healing by microscopy imaging; rate of wound healing is presented by the ratio (%) of wounded areas at each indicated time point to its initial wounded area at time 0 as described in the Additional file 1: Supplemental Methods. **F** Representative images of the same wound at 0 and 30 h are presented. Results represent the means ± SD. The two-tailed Student’s t-test was used for statistical analyses of two-group comparisons. *P < 0.05 and **P < 0.01 versus controls

The IGF signalling pathway is known to stimulate intestinal epithelial proliferation and promote injury recovery of IECs [39]. To investigate whether CREBH is involved in IGF-mediated epithelial cell proliferation and wound repair, mRNA level of IGF1 receptor (IGF1R) was determined in the colons of WT and KO mice with or without DSS treatment. Depletion of CREBH significantly downregulated IGF1R mRNA (Fig. 4A). DSS treatment also suppressed expression of IGF1R gene in the WT mice (Fig. 4A). In vitro, the expression of active CREBH in IPEC-J2 or Caco2 cells enhanced mRNA expression of IGF1R (Fig. 4B; Additional file 1: Fig. S4A) which was abrogated by treating the CREBH-transfected Caco2 cells with TNFα (Additional file 1: Fig. S4B). Further investigating the circulating level of growth hormone IGF1 revealed that plasma IGF1 was significantly lower in the CREBH-KO mice compared to WT (Fig. 4C). Interestingly, *A. muciniphila* treatment significantly upregulated IGF1 in the plasma of WT but not the CREBH-KO mice (Fig. 4C). Administration of *A. muciniphila* further upregulated the mRNA level of IGF1R in the DSS-treated WT mice, a phenotype that was abolished in the CREBH-KO mice (Fig. 4D), indicating the crucial role of CREBH in mediating intestinal IGF signalling and the positive impact of *A. muciniphila* on IGF1 signalling.

It has been well accepted that, following the injury, the intestinal epithelium undergoes a wound healing process which is dependent on the precise balance among migration, proliferation, and differentiation of the epithelial cells adjacent to the wounded area [47]. To further investigate the positive impact of CREBH on intestinal epithelial regeneration, a wound healing assay was performed using Caco2 cells transfected with an empty vector (mock) or CREBH followed by the standard protocol described in the Additional file 1. Images of the wounded area were captured at 0, 3-, 6-, 9-, 24-, 30-, and 48 h as they healed over the time course. The area of circular wounds was quantified by Image J and shown in Fig. 4E. Expression of CREBH significantly enhanced Caco2 cell wound repair compared to the mock transfected cells at 24-, 30-, and 48-hrs (Fig. 4E, F; Additional file 1: Fig. S4C), indicating that overexpression of CREBH stimulates IEC proliferation and wound repair.

### miR-143/-145 induced by *A. muciniphila* target IGFBP5 in IGF signalling and enhances intestinal epithelial regeneration

**Fig. 5:**
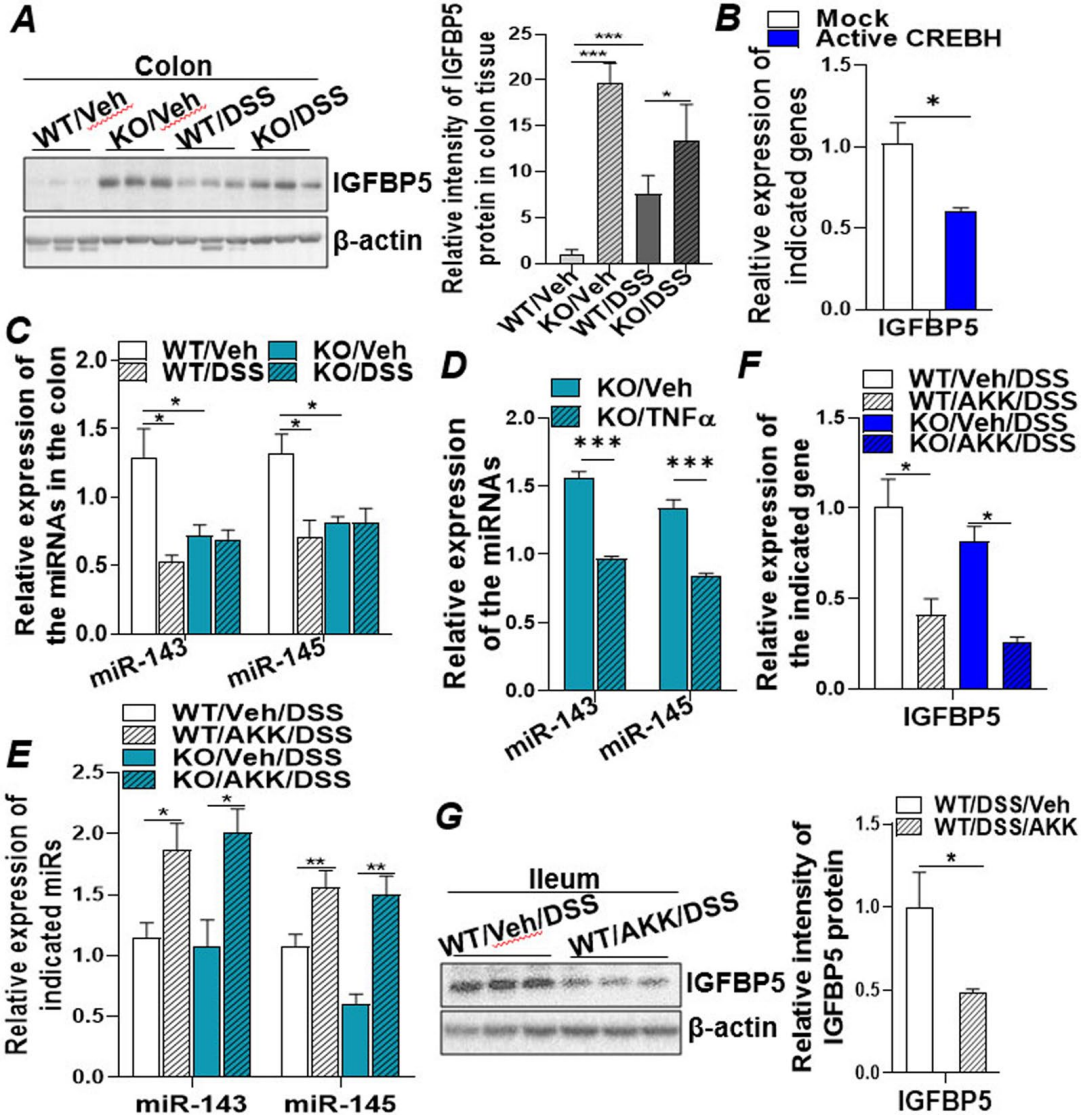
miR-143/-145 induced by *A. muciniphila* target IGFBP5 in IGF signalling and enhances intestinal epithelial regeneration. Four groups of WT and CREBH-KO mice (n = 5–10/groups) were treated with either DSS or Veh (H_2_O) as described in "Methods". Colon and ileum tissues were collected for protein and RNA extractions for the following analysis. **A** Immunoblotting analysis of IGFBP5 and loading control of β-actin in the colon tissues of these mice. **B** Caco2 cells were transfected with mock (empty vector) or an active CREBH (N-terminal CREBH, constitutively active) plasmid for 48 h. Total RNAs were extracted for measurement of mRNA expression of IGFBP5 by qRT-PCR. **C** Expression of miR-143 and miR-145 in the colon tissue of DSS treated mice measured by qRT-PCR and normalized to small nucleolar RNA202. **D** Expression of miR-143 and miR-145 in the ileum tissues of CREBH-KO mice treated with either TNFα (60 ng/g body weight) or vehicle (Veh, saline) for 5 h (n = 5–6/group). **E–G** Four groups of the mice (n = 5–10/groups) were pre-treated with *A. muciniphila* (AKK) or Veh (PBS) followed by Veh (H_2_O) or DSS treatment as described in the "Methods". **E** Expression of miR-143 and miR-145 in the colon tissue measured by qRT-PCR and normalized to small nucleolar RNA202. **F** mRNA expression of IGFBP5 in the colon tissues of the treated mice. **G** Immunoblotting analysis of IGFBP5 and β-actin in ileum tissues of the indicated mice. Results represent the means ± SD. The two-tailed Student’s t-test was used for statistical analyses of two-group comparisons. *P < 0.05 and **P < 0.01 versus controls

To further determine the regulatory mechanism of CREBH on the IGF system, the IGF downstream signalling molecules IGFBPs were investigated. IGFBPs bind to the growth factors, IGFs, with high affinity and sequester the ligand from interaction with IGF receptors [48]. Immunoblotting analysis of IGFBP5 in the colons of DSS-treated WT and CREBH-KO mice revealed that knockout of CREBH or DSS treatment upregulated expression of IGFBP5 (Fig. 5A), whereas gene expression of other IGFBP family members IGFBP1 and IGFBP4 were downregulated under the same pathological condition (Additional file 1: Fig. S5A). Furthermore, overexpression of the active CREBH in Caco2 cells via plasmid transfection suppressed mRNA expression of IGFBP5 but either upregulated IGFBP4 or unaltered IGFBP1 mRNA levels (Fig. 5B; Additional file 1: Fig. S5B), indicating that IGFBP5 is the key IGFBP member that negatively regulates the IGF pathway in this context.

MicroRNAs (miRNAs) are small non-coding RNAs that regulate multiple cellular processes by inducing decay and/or translational repression of the target mRNA [49, 50]. miRNAs have been validated to target more than 60% of mRNAs in the genome and are fundamentally involved in diverse pathophysiological processes, including IBD [51–53]. To investigate if miRNAs are involved in the upregulation of IGFBP5 in CREBH-KO and DSS-treated mice, we searched for miRNAs that can potentially target the 3′-UTR of IGFBP5. Bioinformatics analysis revealed that miR-143-3p and miR-203-3p may potentially interact with two binding sequences within the 3′-UTR of IGFBP5 (Additional file 1: Fig. S5C). The targeted sequences are highly conserved among vertebrates, including humans, rats, and mice, implicating the evolutional importance of these target sites. MiR-143 is located in close proximity with miR-145 on the encoding genes where they are co-transcribed in the same bicistronic transcript cluster [54]. Examining the expression of miR-143/145 cluster in the colons of CREBH-KO and DSS-treated mice revealed the significant downregulation of miR-143/145 by depletion of CREBH or DSS treatment (Fig. 5C). Treatment of these mice with TNFα further uncovered the inhibitory effect of inflammatory cytokine TNFα on intestinal miR-143/145 expression but not on miR-203-3p in the KO mice (Fig. 5D; Additional file 1: Fig. S5D), implicating the specific role of miR-143/145 in the intestinal inflammation. Treatment with *A. muciniphila* restored the expression of miR-143/145 in the colons of DSS treated WT and KO mice (Fig. 5E), which was associated with the decreased mRNA and protein of IGFBP5 (Fig. 5F, G), demonstrating that the elevated miR-143 targeted IGFBP5 mRNA and inhibited protein translation. In vitro, incubation of Caco2 cells with TNFα (20 ng/mL) induced expression of IGFBP5 (Additional file 1: Fig. S5E), which can be inhibited by expressing CREBH in the TNFα-treated cells (Additional file 1: Fig. S5F). Together, these data demonstrate that downregulation of miR-143 induced by DSS or depletion of CREBH releases their inhibition on IGFBP5, which subsequently targets the IGF mediated intestinal epithelial regeneration and wound repair. 

### An outer membrane protein of *A. muciniphila* Amuc_1100 coupled with CREBH to regulate IEC proliferation and gut barrier integrity

**Fig. 6:**
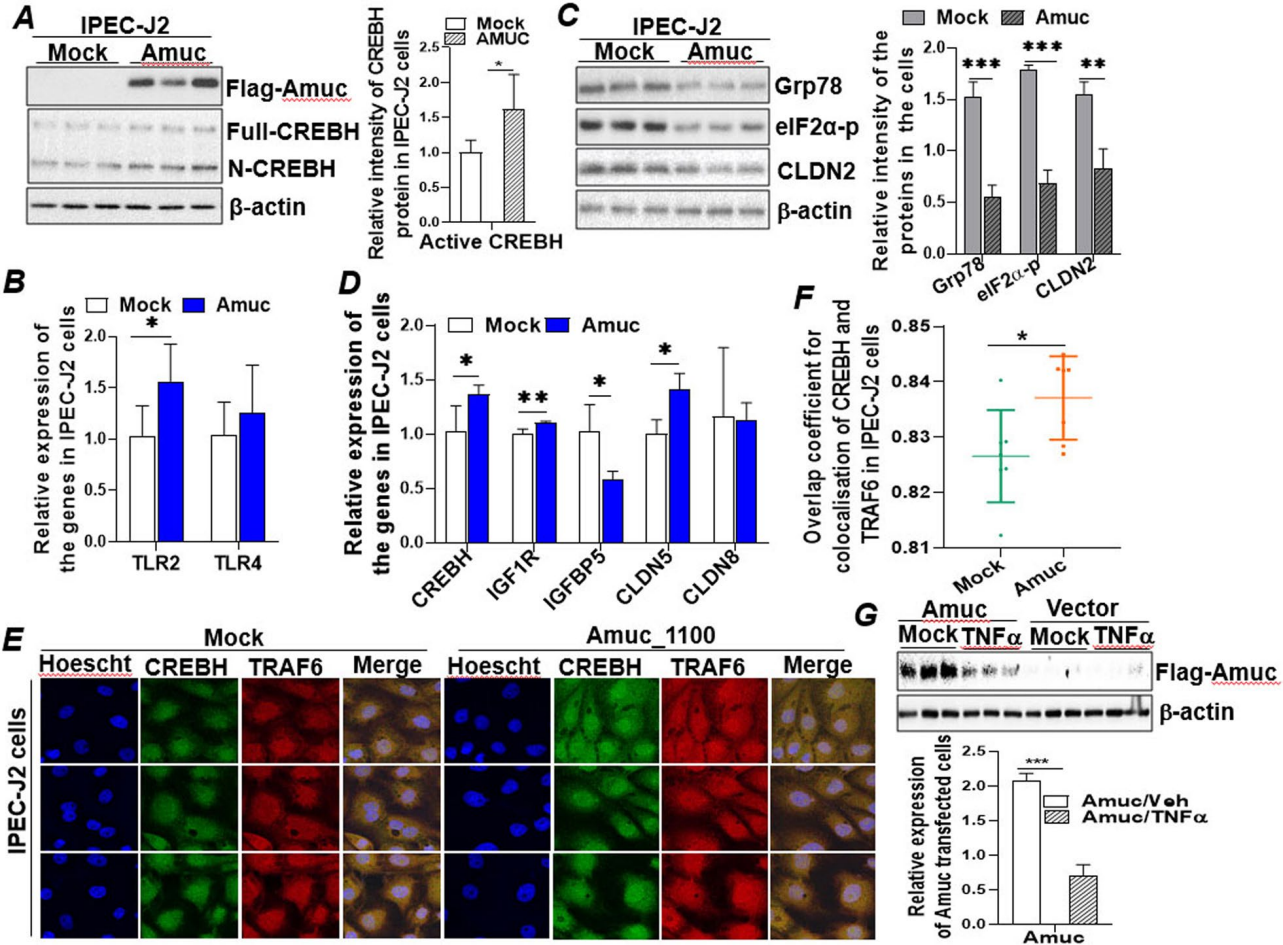
An outer membrane protein of *A. muciniphila* Amuc_1100 coupled with host CREBH to regulate IEC proliferation and gut barrier integrity. IPEC-J2 or Caco2 cells were transfected with empty vector (pcDNA3.1) or vector expressing Amuc_1100 (Amuc) for 48 h. Total mRNA or protein was extracted for the following analyses: **A** Immunoblot analysis of Amuc_1100 (Amuc), Full-length CREBH, N-terminal CREBH (N-CREBH, active form), and β-actin. **B** mRNA expression of TLR2 and TLR4. **C** Immunoblot analysis of Grp78, eIF2α-p, CLDN2, and β-actin. **D** mRNA expression of CREBH, IGF1R, IGFBP5, CLDN5, and CLDN8 in the transfected IPEC-J2 cells. **E** Immunofluorescent confocal images of the transfected IPEC-J2 cells immune-stained with anti-CREBH and anti-TARF6 antibodies, and Hoescht 33,349 as described in the Additional file 1: Supplemental Methods. **F** Overlap coefficient analysis of the immunofluorescence images in **E**, showing co-localisation of CREBH and TRAF6. **G** Immunoblot analysis of Amuc_1100 (Amuc) and β-actin expression in Caco2 cells transfected with empty vector or Amuc_1100 (Amuc) expressing vector (for 48 h) treated with or without TNFα (20 ng/mL) for 24 h. Results represent the means with SD. The two-tailed Student’s t-test was used for statistical analyses of two-group comparisons. *P < 0.05 and **P < 0.01 versus controls

To explore the potential of developing gut bacterial component as therapeutic approach for human gut diseases (i.e., IBD), we searched for bacterial component(s) that can recapitulate the health beneficial effect of *A. muciniphila.* The outer membrane protein *A. muciniphila*, Amuc_1100, is one of the most abundant membrane proteins that presents on a gene cluster related to pilus formation, which may suggest its potential to interact with host intestinal epithelial cells and engage in host metabolism [55]. To investigate if Amuc_1100 can be expressed in mammalian IECs and regulates the intestinal cellular activities, we cloned the translational open reading frame of Amuc_1100 from *A. muciniphila* into a mammalian cell expression vector pcDNA3.1-Flag as detailed in the "Methods". Successful expression of Amuc_1100 protein in IPEC-J2 cells was verified by an immunoblotting analysis (Fig. 6A). More importantly, expression of Amuc_1100 upregulated and activated cellular CREBH in the transfected cells, indicated by the increased active form of CREBH (N-CREBH) (Fig. 6A). TLR (TLR2 and TLR4) signalling has been reported to be the key mediators of commensal bacteria in maintaining host intestinal epithelial homeostasis. Activation of TLRs by either the TLR2 or TLR4 ligand is critical for the protection against gut injury and associated mortality [28]. Consistent with this view, we found that expression of Amuc_1100 stimulated mRNA expression of TLR2 but not TLR4 in IPEC-J2 cells (Fig. 6B), which was accompanied by less activation of ER stress markers, Glucose related protein 78 (Grp78) and phospho-eIF2α, and inhibition of the leaky gut tight junction, CLDN2 (Fig. 6C). Inhibition of CLDN2 was further observed in the Amuc_1100 transfected Caco2 cells (Additional file 1: Fig. S6A). Moreover, Amuc_1100 stimulated mRNA expression of CREBH, IGF1R, and CLDN5 but suppressed IGFBP5 in the IPEC-J2 cells whereas mRNA of CLDN8 remained unchanged (Fig. 6D). These data demonstrated that Amuc_1100 is capable of recapitulating the health beneficial effect of *A. muciniphila* in IECs, likely via mediation of CREBH and TLR2.

We further investigate the mechanism by which Amuc_1100 activates CREBH. The TNF receptor associated factor 6 (TRAF6), a molecule mediating TLR singling, has been shown to interact with CREBH and facilitate its cleavage and activation in mouse liver challenged with bacterial endotoxin lipopolysaccharide (LPS) [56]. To examine if TRAF6 is involved in the activation of CREBH induced by Amuc_1100, an immunostaining assay followed by confocal imaging analysis was performed. Consistent with the data obtained by immunoblotting analysis in Fig. 6A, immunostaining analysis revealed that expression of Amuc_1100 in IPEC-J2 cells activated CREBH, indicated by the increased nuclear localization of CREBH (active CREBH) (Fig. 6E). Overlap coefficient analysis revealed that Amuc_1100 promoted co-localization of CREBH with TRAF6 which may facilitate the cleavage and activation of CREBH (Fig. 6F). More intriguingly, treating the Amuc_1100 transfected cells with TNFα inhibited expression of Amuc_1100 and abrogated the positive regulation of Amuc_1100 on CREBH, CLDN5 and IGF1R but restored IGFBP5 mRNA expression (Fig. 6G; Additional file 1: Fig. S6B). Together, these results demonstrate that the bacterial membrane protein Amuc_1100 can be expressed in porcine and human IECs and recapitulate the health beneficial effects of *A. muciniphila* on gut barrier integrity and IGF signalling via mediation of TLR2 and CREBH.

## Discussion

**Fig. 7:**
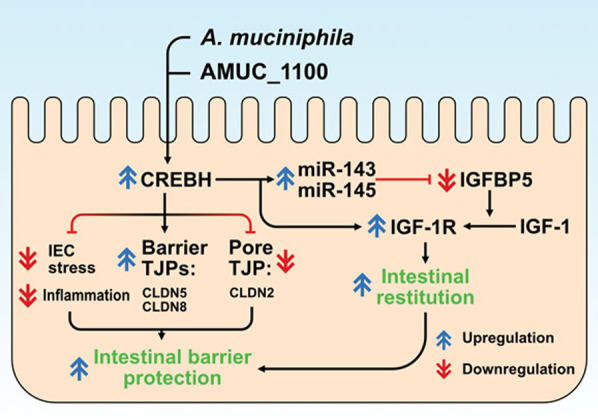
* A. muciniphila* and its membrane protein Amuc_1100 enhances intestinal CREBH expression which in turn ameliorates colonic ER stress and inflammation induced by DSS and promotes intestinal barrier integrity by upregulating expression of protective gut tight junctions (CLDN5 and CLDN8) but inhibiting the leaky gut mediator, CLDN2. CREBH further coupled with microRNA-143/145 to positively regulate the IGF-1 signalling pathway involved in intestinal epithelial regeneration and wound repair. An intact CREBH is essential to mediate the beneficial effect of *A. muciniphila* and its membrane protein Amuc_1100 in gut health

Our study uncovers a key link between *A. muciniphila* and intestinal CREBH in gut health that yields several novel findings (Fig. 7). First, increased colonization of *A. muciniphila* in the GI tract ameliorates ER stress in the IECs, improves gut barrier integrity, and reduces metabolic inflammation via CREBH. Second, increased CREBH induced by *A. muciniphila* coupled with miR143/145 to enhance IGF signalling in intestinal epithelial regeneration and wound healing. Third, the newly identified outer membrane protein of *A. muciniphila*, Amuc_1100, can be expressed in mammalian cells and recapitulate the health beneficial effect of *A. muciniphila* in IECs, at least partially, which may lend support to pharmaceutical development focusing on gut bacterial components (e.g., membrane proteins) instead of whole bacteria for the treatment of IBD and other human diseases. In summary, novel findings from this study provide advanced insights into the mutual interaction between host gene regulation and gut microbiome in intestinal barrier homeostasis and epithelial regeneration via CREBH and miRNAs, which may shed light on exploring therapeutic strategies for the treatment of inflammatory bowel disorders.

Dysbiosis plays a pivotal role in the pathogenesis of human IBD [57]. Previously, administration of *A. muciniphila* was reported to restore the intestinal thickness of the inner mucus layer in high-fat diet induced obese mice and therefore contribute to the prevention of dietary induced metabolic disease [11]. Regulation of *A. muciniphila* on the host gene expression has been studied in (mono-associated) mice and organoids, which identified that most of the genes affected by the gut bacteria were involved in immune and metabolic responses [6, 58, 59]. In this study, we demonstrated that CREBH, miR-143/145, and IGF system are key mediators in the crosstalk between host intestinal homeostasis and gut bacteria. Upregulation of CREBH by *A. muciniphila* ameliorates ER stress in IECs, enhances expression of protective gut tight junctions, promotes IEC proliferation and inhibits gut leakage. These novel findings decipher an underlying mechanism for why decreased *A. muciniphila* abundance contributes to the development of human ulcerative colitis and inflammatory bowel disorders [13, 60–63]. 

A fine balance between tight junction proteins is critical for the maintenance of paracellular integrity in the gut. Functional loss of epithelial cell barrier integrity is thought to be the initial pathological change that underlies injury and inflammation of IBD as this results in the migration of luminal antigens into the submucosa, exposing lamina propria immune cells to these antigens and eliciting inflammatory response in the intestine [64]. Compromised intestinal barrier integrity and increased IEC permeability allows bacteria-derived molecules (e.g., LPS) to leak into the mucosa and initiate inflammatory responses. Clinically, altered expression of tight junction proteins and increased epithelial permeability preceding disease relapse are observed in patients with Crohn’s disease, stressing the essential role of tight junction proteins in IBD [65, 66]. In this study, we found that DSS-induced IBD resulted in robust increase of plasma endotoxin (LPS), which can be inhibited by in vivo administration of *A. muciniphila* despite that *A. muciniphila* is a Gram-negative gut bacterium that contains LPS in its outer membrane. Depletion of CREBH abrogates the protective effect of *A. muciniphila* on DSS-induced gut permeability, resulting in increased plasma LPS in the CREBH-KO mice. Mechanistic study further demonstrated the positive regulation of CREBH on IEC’s regeneration and wound repair via modulating the IGF signaling and miRNA-143/145. These novel findings strongly support that an intact CREBH is essential for maintaining a healthy balance between gut integrity and permeability.

In contrast to the studies that show the health beneficial effect of *A*. *muciniphila* on intestinal barrier integrity, several mouse studies have reported that increased numbers of the mucosal resident bacteria were found in DSS-induced colitis [67–69]. Ganesh et al. also reported that administration of *A. muciniphila* exacerbated *Salmonella enterica Typhimurium* (*S. Typhimurium*)-induced intestinal inflammation in a gnotobiotic mouse model [70]. The possible mechanisms behind these observations could be that the increased mucosa-associated bacteria found in IBD is needed to produce more nutrients to sustain non-mucolytic mucosa-associated bacteria upon IBD. Furthermore, although increased total mucosa-associated bacterial 16 S rRNA genes were found in the intestinal epithelium of patients with Crohn’s disease or ulcerative colitis, the bacterial abundance are disproportionally increased in some mucolytic bacteria, including *Ruminococcus gnavus* and *Ruminococcus torques* but decreased in *A. muciniphila* [13]. Future studies to delineate the underlying mechanisms are needed to address the role of *A. muciniphila* and other mucosa-associated bacteria in modulating intestinal barrier integrity.

*A. muciniphila* has been identified as a unique gut bacterium in the field of next-generation probiotic research because of its colonization in the intestinal mucus layer, an interface close to host intestinal cells and other gut bacteria, as well as its health beneficial effects on several pathological conditions, including obesity, type-2 diabetes, atherosclerosis, and gut disorders [11, 14–16]. However, the great hurdle in the translational application of *A. muciniphila* in clinical therapeutics is its high sensitivity to oxygen and its growth medium containing animal-derived nutrients that is not compatible for human administration. To circumvent this obstacle, scientists have tried to explore and isolate bioactive components within *A. muciniphila* that can recapitulate the health beneficial function of the whole bacteria. Previously, Plovier et al. cloned an outer membrane protein of *A. muciniphila*, Amuc_1100, into a bacterial expression vector and expressed the protein in *E. coli.* The purified protein was then delivered to the high-fat diet-induced obese mice via oral gavage and was found that Amuc_1100 protein interacted with TLR2, and partially recapitulates the function of *A. muciniphila* against obesity and insulin resistance [17]. Amuc_1100 is a highly abundant pili-like membrane protein that localizes at the outside of *A. muciniphila*, a location that may facilitate its crosstalk with host enterocytes. As discussed above, CREBH is also presented in the intestinal epithelial cells of the villi [27], the physical proximity between Amue_1100 and CREBH in the gut may facilitate their interaction with each other and exert their health beneficial effect in the gut. Isolation and identification of bioactive components (i.e., Amuc_1100) from gut bacteria that is capable of recapitulating all or part of the health beneficial effects of the live organism could be an innovative strategy for the therapeutics of metabolic diseases. In this study, we successfully expressed a membrane protein of *A. muciniphila* Amuc_1100 in porcine intestinal epithelial cell IPEC-J2 and human Caco2 cells, and further demonstrated that the presence of Amuc_1100 in mammalian IECs activated CREBH, which subsequently mitigated IEC’s ER stress and stimulated the expression of protective tight junction proteins, epithelial regeneration and wound repair. We further showed that TLR2 and TRAF6 mediated the activation of CREBH in this context. These novel findings may contribute to the development of treatment strategy to utilize *A. muciniphila* or its components to ameliorate human diseases, such as IBD and other inflammatory gut disorders.

## Conclusions

This study uncovers a novel regulatory mechanism that links *A. muciniphila* and its membrane protein Amuc_1100 with host CREBH, IGF signalling and microRNAs in mitigating intestinal inflammatory stress and gut barrier permeability as well as promoting intestinal wound healing. This novel finding may lend support to develop therapeutic approaches for IBD by manipulating the mutual, association between host genes, gut bacteria and its bioactive components.

## Supplementary Information

**Additional file 1**: **Figure S1**.Measurement of disease activity index over a 14-day period in DSS treated mice with or without *A. muciniphila* treatment.Immunohistochemistrystaining of anti-F4/80 antibody in the mouse distal colonic tissues as described in the Additional file 1: Supplemental Methods. Results represent the means±SD. The two-tailed Student’s t-test was used for statistical analyses of two-group comparisons. *P < 0.05 and **P < 0.01 versus controls.>. **Figure S2**.qRT-PCR analysis of CREBH mRNA expression in the ileum of WT micepre-treatment with *A. muciniphila*or Vehfollowed by DSStreatment for 14 days as described in the Methods.Body weights of the CREBH-KO micepre-treated with *A. muciniphila*or Vehfollowed by DSStreatment for 14 days as described in the Methods. Results represent the means±SD. The two-tailed Student’s t-test was used for statistical analyses of two-group comparisons. *P < 0.05 and **P < 0.01 versus controls.>. **Figure S3**.qRT-PCR analysis of mRNA expression of CLDN2 in Caco2 cells treated with TNFαfor 12 and 24 hrs, respectively.qRT-PCR analysis of mRNA expression of CLDN2 in Caco2 cells treated with IL-1βfor 24 and 48 hrs, respectively.qRT-PCR analysis of mRNA expression of CLDN5, CLDN8, OCLN, and ZO-1 in Caco2 cells transfected with mock vectoror vector expressing constitutively active CREBHfor 48 hrs.qRT-PCR analysis of mRNA expression of CLDN5, CLDN8, OCLN, and ZO-1 in Caco2 cells transfected mock or constitutively active CREBHfor 48 hrs and treated with or without TNFαfor 24 hrs. Results represent the means ± SD. The two-tailed Student’s t-test was used for statistical analyses of two-group comparisons. *P < 0.05 and **P<0.01 versus controls. >. **Figure S4**.qRT-PCR analysis of mRNA expression of IGF1R in Caco2 cells transfected with mock vectoror vector expressing constitutively active CREBHfor 48 hrs.qRT-PCR analysis of mRNA expression of IGF1R in Caco2 cells transfected mock or constitutively active CREBHfor 24 h followed by additional 24 h treatment with TNFα.The non-outlined microscopy images of the wounded Caco2 cell monolayer shown in Figure 4F. Results represent the means ± SD. The two-tailed Student’s t-test was used for statistical analyses of two-group comparisons. **P < 0.01 versus controls. >. **Figure S5**.qRT-PCR analysis of mRNA expression of IGFBP1 and IGFBP4 in the colon tissues of the WT and CREBH-KO mice treated with DSSor vehiclein drinking water for 14 days.qRT-PCR analysis of mRNA expression of IGFBP1 and IGFBP4 in Caco2 cells transfected with mock vectoror vector expressing constitutively active CREBHfor 48 hrs.miR-143-3p and miR-203-3p response elements within the 3’- UTR of human, rat and mouse IGFBP5 mRNA predicted by TargetScan 7.2.Expression of miR-203-3p in the ileums of WT and CREBH-KO mice treated with either Vehor TNFα for 5 hrs.mRNA expression of IGFBP5 in Caco2 cells treated with mockor TNFαfor 24 hrs.qRT-PCR analysis of mRNA expression of IGFBP5 in Caco2 cells transfected with mock vectoror vector expressing constitutively active CREBHfor 48 h and treated with or without TNFαfor 24 hrs. Results represent the means with SD. The two-tailed Student’s t-test was used for statistical analyses of two-group comparisons. *P < 0.05 versus controls. **Figure S6**.Immunoblot analysis of CLDN2 and β-actin in Caco2 cells transfected with mock vectoror vector expressing Amuc_1100 for 48 hrs.mRNA expression of CREBH, CLDN5, CLDN8, IGF1R, and IGFBP5 in Caco2 cells transfected with mock vectoror vector expressing constitutively active CREBHfor 48 h and treated with or without TNFαfor 24 hrs. Results represent the means with SD. The two-tailed Student’s t-test was used for statistical analyses of two group comparisons. **P < 0.01 versus controls. >. **Table S1**. Sequences of primers used in this study. **Table S2**. Scoring system for disease activity index.

## Data Availability

The datasets supporting conclusions of this article are available from the corresponding author upon reasonable request.

## Abbreviations

AKK: Akkermansia muciniphila
Caco2: Colonic adenocarcinoma cells
CD: Crohn's disease
CLDN: Claudin
CREBH: Cyclic adenosine monophosphate responsive element binding protein H
DAI: Disease assessment index
DSS: Dextran sulfate sodium
eIF2a: Eukaryotic translation initiation factor 2a
ER: Endoplasmic reticulum
FITC-dextran: Fluorescein Isothiocyanate-Dextran
GRP78: Glucose related protein 78
HNF: Hepatocyte nuclear factor
IBD: Inflammatory bowel disease
IEC: Intestinal epithelial cell
IGF: Insulin-like growth factor
IGF1R: Insulin-like growth factor receptor 1
IGFBP: Insulin-like growth factor binding protein
IL: Interleukin
IPEC-J2: Porcine small intestinal epithelial cells
JAM: Junctional adhesion molecule
JNK: c-Jun N-terminal kinase
KO: Knock out
LPS: Lipopolysaccharide
miR: microRNA
mRNA: Messenger RNA
NFkB: Nuclear factor kappa B
OCLN: Occludin
TLR: Toll-like receptor
TNFa: Tumour necrosis factor a
TRAF6: TNF receptor associated factor
Veh: Vehicle
ZO: Zonnula occludens
