# Supplementary material for: Akkermansia muciniphila and its membrane protein ameliorates intestinal inflammatory stress and promotes epithelial wound healing via CREBH and miR-143/145
Source: J Biomed Sci. 2023 Jun 7;30:38. doi: 10.1186/s12929-023-00935-1 (PMC10249216; doi:10.1186/s12929-023-00935-1)
Supplement: Supplementary file 1 — Additional file 1: Figure S1.Measurement of disease activity index over a 14-day period in DSS treated mice with or without A. muciniphila treatment.Immunohistochemistrystaining of anti-F4/80 antibody in the mouse distal colonic tissues as described in the Additional file 1: Supplemental Methods. Results represent the means±SD. The two-tailed Student’s t-test was used for statistical analyses of two-group comparisons. *P < 0.05 and **P < 0.01 versus controls.>. Figure S2.qRT-PCR analysis of CREBH mRNA expression in the ileum of WT micepre-treatment with A. muciniphilaor Vehfollowed by DSStreatment for 14 days as described in the Methods.Body weights of the CREBH-KO micepre-treated with A. muciniphilaor Vehfollowed by DSStreatment for 14 days as described in the Methods. Results represent the means±SD. The two-tailed Student’s t-test was used for statistical analyses of two-group comparisons. *P < 0.05 and **P < 0.01 versus controls.>. Figure S3.qRT-PCR analysis of mRNA expression of CLDN2 in Caco2 cells treated with TNFαfor 12 and 24 hrs, respectively.qRT-PCR analysis of mRNA expression of CLDN2 in Caco2 cells treated with IL-1βfor 24 and 48 hrs, respectively.qRT-PCR analysis of mRNA expression of CLDN5, CLDN8, OCLN, and ZO-1 in Caco2 cells transfected with mock vectoror vector expressing constitutively active CREBHfor 48 hrs.qRT-PCR analysis of mRNA expression of CLDN5, CLDN8, OCLN, and ZO-1 in Caco2 cells transfected mock or constitutively active CREBHfor 48 hrs and treated with or without TNFαfor 24 hrs. Results represent the means ± SD. The two-tailed Student’s t-test was used for statistical analyses of two-group comparisons. *P < 0.05 and **P<0.01 versus controls. >. Figure S4.qRT-PCR analysis of mRNA expression of IGF1R in Caco2 cells transfected with mock vectoror vector expressing constitutively active CREBHfor 48 hrs.qRT-PCR analysis of mRNA expression of IGF1R in Caco2 cells transfected mock or constitutively active CREBHfor 24 h followed by additional 24 [file 12929_2023_935_MOESM1_ESM.pdf]

Additional File

Akkermansia muciniphila and its Membrane Protein Ameliorates Intestinal Inflammatory Stress and Promotes Epithelial Wound Healing via CREBH and miR-143/145

Henry Wade[1,§], Kaichao Pan[2,§], Qihua Duan[1], Szczepan Kaluzny[1], Ekta Pandey[3], Linda Fatumoju[3], Viswanathan Saraswathi[4], Rongxue Wu[2], Edward N. Harris[3] and Qiaozhu Su[1*]

[1]Institute for Global Food Security, School of Biological Sciences, Queen's University Belfast, Belfast BT9 5DL, United Kingdom

[2]Department of Medicine, Section of Cardiology, University of Chicago, Chicago, USA

[3]Department of Biochemistry, University of Nebraska-Lincoln, Lincoln, NE, 68583, USA

[4]University of Nebraska Medical Center, Omaha, NE, 68198, USA

[§]**These authors contribute equally to the work.**

Corresponding author Email: q.su@qub.ac.uk

**Contents**	**Pages**
Figure S1.	S2
Figure S2.	S3
Figure S3.	S4
Figure S4.	S5
Figure S5.	S6
Figure S6.	S7
Supplemental Methods	S8-9
Supplemental Table 1. Sequences of primers used in this study	S9-10
Supplemental Table 2. Scoring system for disease activity index	S11

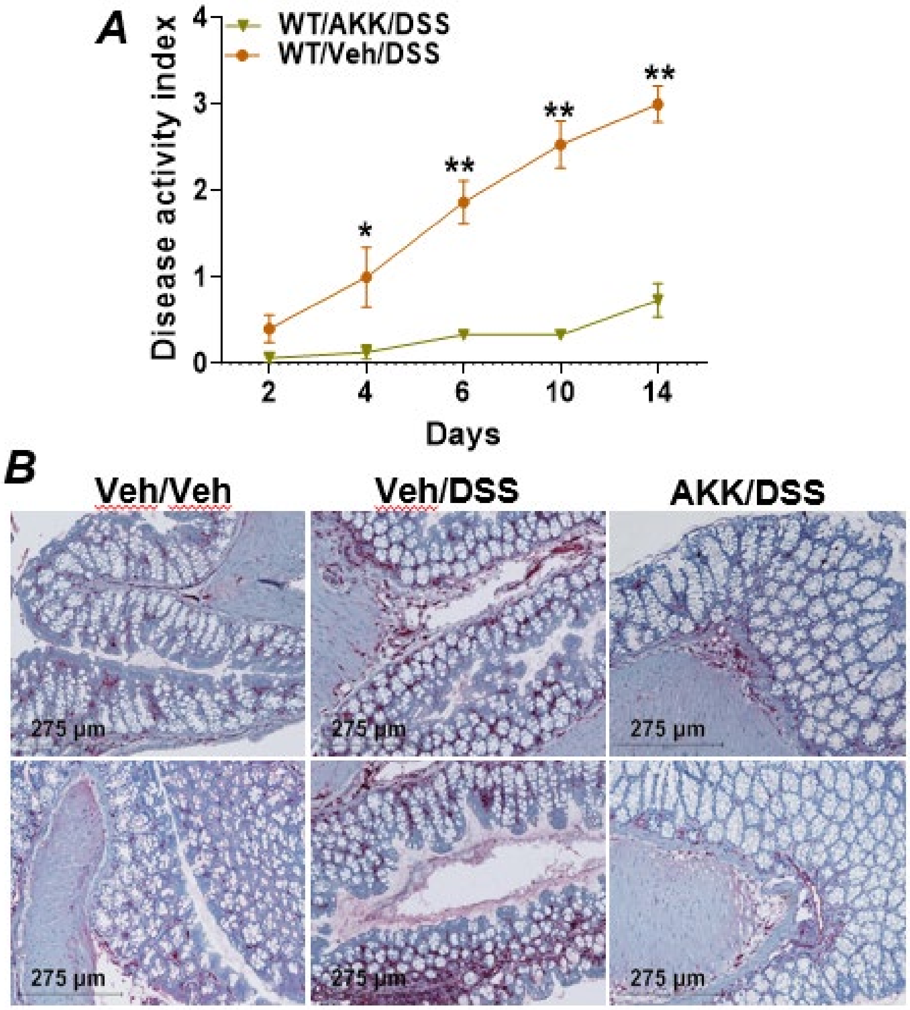

Figure S1. (**A**) Measurement of disease activity index over a 14-day period in DSS treated mice with or without *A. muciniphila* treatment. (**B**) Immunohistochemistry (IHC) staining of anti-F4/80 antibody in the mouse distal colonic tissues as described in the Additional File: Supplemental Methods. Results represent the means±SD. The two-tailed Student's t-test was used for statistical analyses of two-group comparisons. *P < 0.05 and **P<0.01 versus controls.

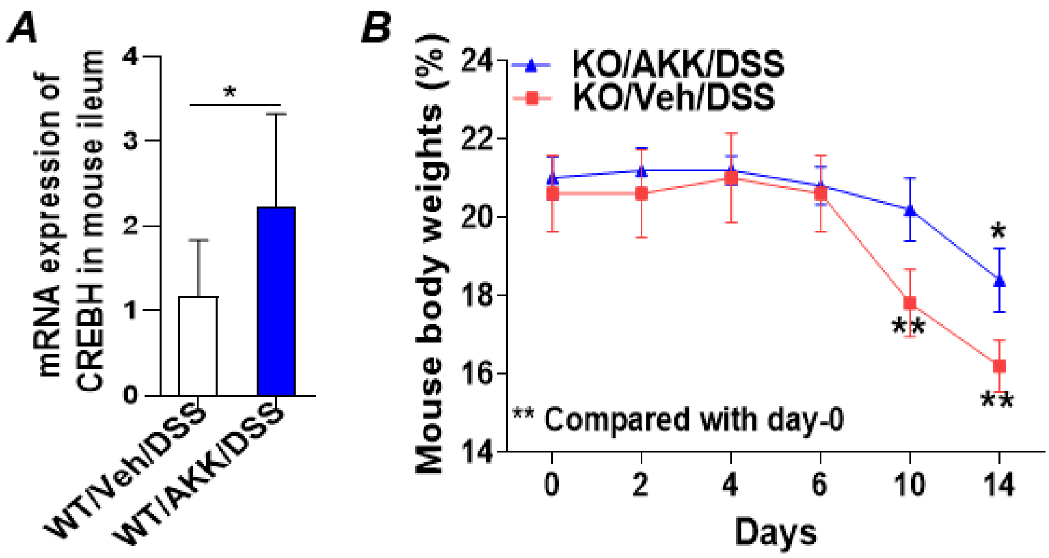

Figure S2. (A) qRT-PCR analysis of CREBH mRNA expression in the ileum of WT mice (n=5-10/groups) pre-treatment with *A. muciniphila* (AKK) or Veh (PBS) followed by DSS (1.5%) treatment for 14 days as described in the Methods. **(B)** Body weights of the CREBH-KO mice (n=5-10/groups) pre-treated with *A. muciniphila* (AKK) or Veh (PBS) followed by DSS (1.5%) treatment for 14 days as described in the Methods. Results represent the means±SD. The two-tailed Student's t-test was used for statistical analyses of two-group comparisons. *P < 0.05 and **P<0.01 versus controls.

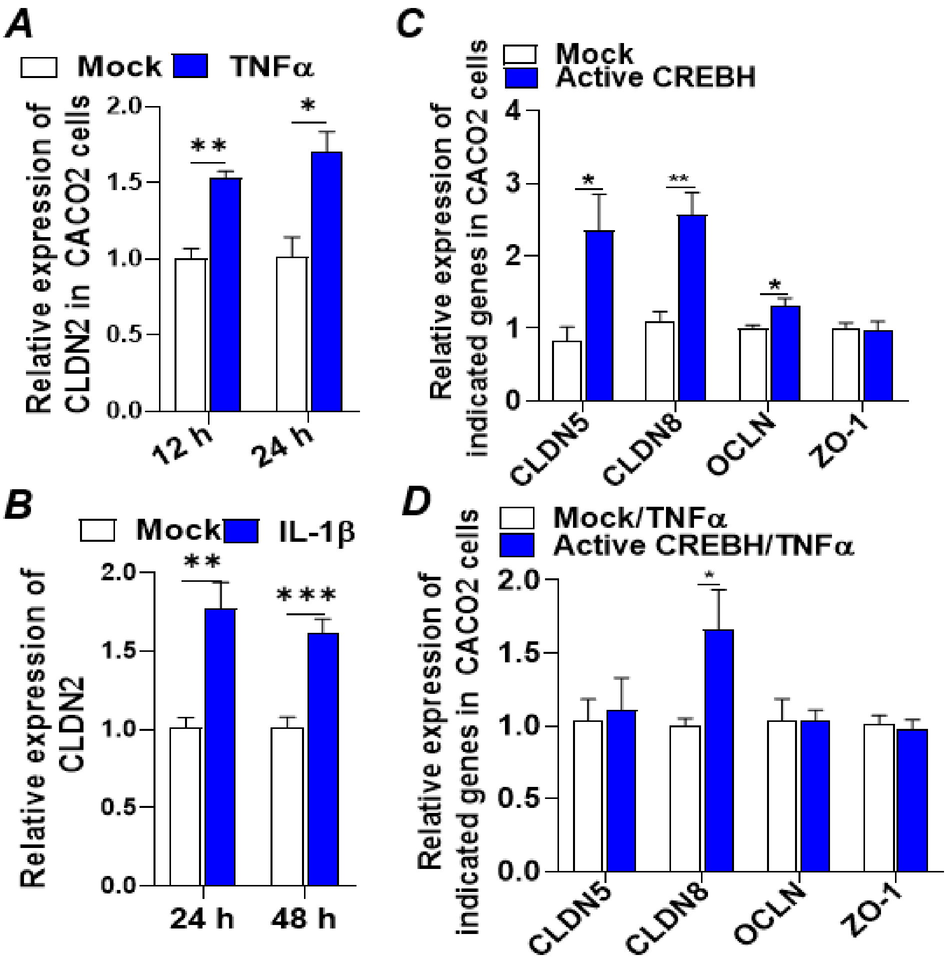

Figure S3. (**A**) qRT-PCR analysis of mRNA expression of CLDN2 in Caco2 cells treated with TNFα (20 ng/mL) for 12 and 24 hrs, respectively. (**B**) qRT-PCR analysis of mRNA expression of CLDN2 in Caco2 cells treated with IL-1β (20 ng/mL) for 24 and 48 hrs, respectively. (**C**) qRT-PCR analysis of mRNA expression of CLDN5, CLDN8, OCLN, and ZO-1 in Caco2 cells transfected with mock vector (pFLAG-CMV2) or vector expressing constitutively active CREBH (N-terminal CREBH) for 48 hrs. (**D**) qRT-PCR analysis of mRNA expression of CLDN5, CLDN8, OCLN, and ZO-1 in Caco2 cells transfected mock or constitutively active CREBH (N-terminal CREBH) for 48 hrs and treated with or without TNFα (20ng/mL) for 24 hrs. Results represent the means±SD. The two-tailed Student's t-test was used for statistical analyses of two-group comparisons. *P < 0.05 and **P<0.01 versus controls.

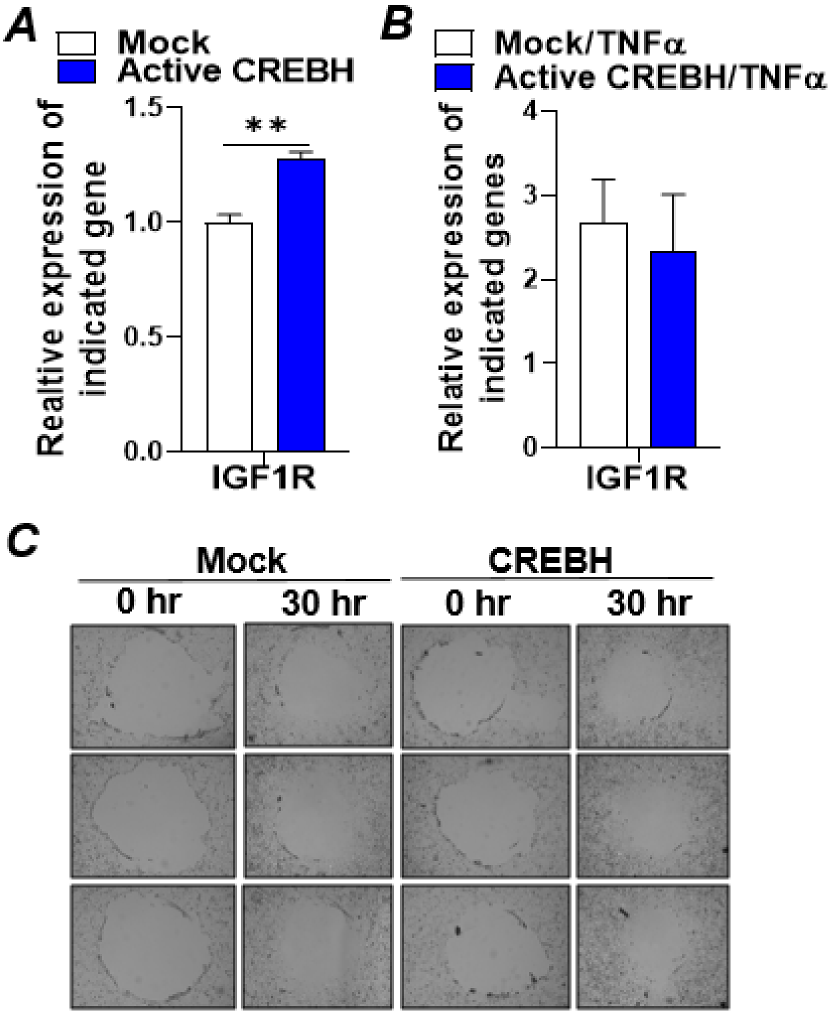

Figure S4. (**A**) qRT-PCR analysis of mRNA expression of IGF1R in Caco2 cells transfected with mock vector (pFLAG-CMV2) or vector expressing constitutively active CREBH (N-terminal CREBH) for 48 hrs. (**B**) qRT-PCR analysis of mRNA expression of IGF1R in Caco2 cells transfected mock or constitutively active CREBH (N-terminal CREBH) for 24 hrs followed by additional 24 hr treatment with TNFα (20 ng/mL). (**C**) The non-outlined microscopy images of the wounded Caco2 cell monolayer shown in Figure 4F. Results represent the means±SD. The two-tailed Student's t-test was used for statistical analyses of two-group comparisons. **P<0.01 versus controls.

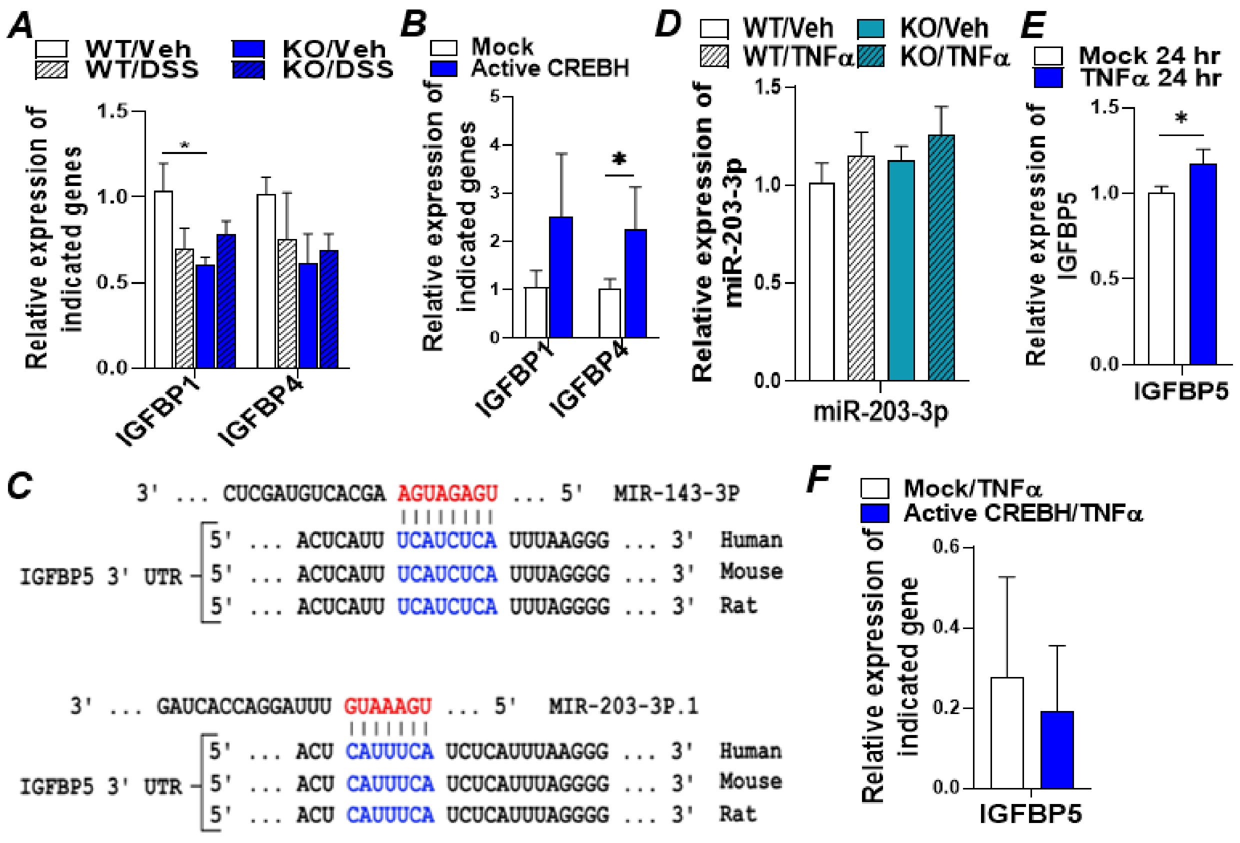

Figure S5. (**A**) qRT-PCR analysis of mRNA expression of IGFBP1 and IGFBP4 in the colon tissues of the WT and CREBH-KO mice treated with DSS (1.5%) or vehicle (Veh, H_2O) in drinking water for 14 days. (**B**) qRT-PCR analysis of mRNA expression of IGFBP1 and IGFBP4 in Caco2 cells transfected with mock vector (pFLAG-CMV2) or vector expressing constitutively active CREBH (N-terminal CREBH) for 48 hrs. (**C**) miR-143-3p and miR-203-3p response elements within the 3'-UTR of human, rat and mouse IGFBP5 mRNA predicted by TargetScan 7.2. (**D**) Expression of miR-203-3p in the ileums of WT and CREBH-KO mice treated with either Veh (Saline) or TNFα for 5 hrs. (**E**) mRNA expression of IGFBP5 in Caco2 cells treated with mock (H_2O) or TNFα (20 ng/mL) for 24 hrs. (**F**) qRT-PCR analysis of mRNA expression of IGFBP5 in Caco2 cells transfected with mock vector (pFLAG-CMV2) or vector expressing constitutively active CREBH (N-terminal CREBH) for 48 hrs and treated with or without TNFα (20ng/mL) for 24 hrs. Results represent the means with SD. The two-tailed Student's t-test was used for statistical analyses of two-group comparisons. *P < 0.05 versus controls.

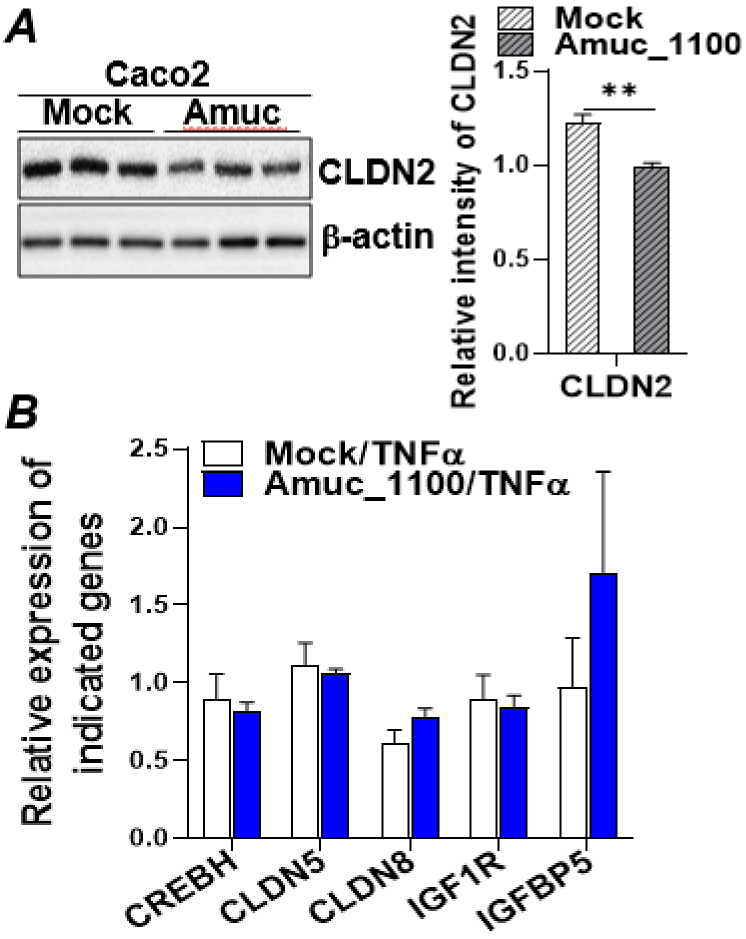

Figure S6. (**A**) Immunoblot analysis of CLDN2 and β-actin in Caco2 cells transfected with mock vector (pcDNA3.1 vector) or vector expressing Amuc_1100 for 48 hrs. (**B**) mRNA expression of CREBH, CLDN5, CLDN8, IGF1R, and IGFBP5 in Caco2 cells transfected with mock vector (pFLAG-CMV2) or vector expressing constitutively active CREBH (N-terminal CREBH) for 48 hrs and treated with or without TNFα (20ng/mL) for 24 hrs. Results represent the means with SD. The two-tailed Student's t-test was used for statistical analyses of two-group comparisons. **P<0.01 versus controls.

Supplemental Methods

Histological analysis and immunohistochemistry (IHC): For histological analysis, colon tissue sections collected from the experimental mice were fixed, dehydrated, embedded and sectioned (5 µm) for H&E staining. For IHC staining, tissue samples were first fixed and frozen in Optimal Cutting Temperature (OCT) compound, mounted in paraffin and the paraffin sections were deparaffinised, endogenous enzymes were inactivated and antigens were thermally repaired. The sections were then blocked and stained with anti-F4/80 (#A700-209 Fortis Life Sciences) antibodies at 1:500 dilution followed detection with the Roche UltraView Alkaline Phosphatase Red Detection Kit (760-501). Stained slides were scanned by an EVOS FL Auto Cell Imaging System.

Immunofluorescent imaging Assay: $1x10^5$ IPEC-J2 cells were seeded onto frosted glass slides (Fisher Scientific: J1800AMNZ) and allowed to incubate for 24-hours in full growth media at 37°C with 5% CO2. Media then replaced with media containing recombinant human TNFα (20 ng/mL) (Invitrogen: PHC3015) and incubated for additional 24-hrs. For transfection, cells were transfected with empty PcDNA3.1 vector or PcDNA3.1 vector expressing Amuc_1100 using Lipofectamine 3000 (Thermofisher, L3000008)/Opti-MEM (Gibco, 31985062); 6-hrs post-transfection, media were replaced with full growth media and further incubated for 24 hrs before replacing with full media containing 0 or 20 ng/mL recombinant human TNFα and incubated for additional 24 hrs. Cells were then washed with PBS and fixed with 4% formaldehyde. Cells were permeabilised with 1% Triton X-100+Saponin. Slides were then incubated at room temperature (RT) for 30 mins in 0.12M glycine/PBS before blocking for 1 hr at RT in 1% BSA/PBS. Slides were washed once with PBS before primary antibody incubation for 1 hr at 37°C followed by overnight incubation at 4°C with 200uL 1% BSA containing 1:750 anti-CREB3L3 (E-2) (Santa Cruz, Sc-377332-S) and 1:300 anti-TRAF6 (D-10) (Santa Cruz, Sc-8409). The next day, slides were washed with PBS and incubated in 1%

BSA containing 1:1000 Alexa-647 anti-mouse and 1:500 Alexa-488 anti-rabbit secondary antibodies for 1 hr at 37°C. 1:10,000 Hoescht 33342 was used to stain nucleus at RT. Slides were mounted using Fluoromount-G (00-4958-02). Confocal microscopy conducted using Leica TCS-SP5 inverted microscope.

IEC wound-healing assay: 1x10^5 Caco2 cells seeded into 12-well plate. Upon reaching 70% confluency, cells were transfected with either empty (2μg/mL) pFLAG-cMV2 vector or pFLAG-cMV2 vector expressing constitutively active CREBH using Lipofectamine 3000 for 6 hrs, after which, media was replaced with Caco2 full growth media and cells were allowed to reach 100% confluency. The confluent Caco2 cells monolayer was scratched using a sterile 2 μL pipette tip (0-hr timepoint). Images were captured using an inverted light microscope at 0, 3-, 6-, 9-, 24-, 30-, 48-, and 60-hour time points. Media was refreshed every 24-hrs. Wound area was quantified using ImageJ and healing rate was determined by calculating the ratio (%) of wounded area at each time point to the wounded area at 0 hr.

Table S1. Sequences of primers used in this study

**Mouse**	**Forward (5`-3`)**	**Reverse (5`-3`)**
**CLDN2**	TGGGCTTTATCCCAGTTGC	TGCTGAGATGATGCCCAAG
**CLDN3**	CATCACGTCGCAGAACATCT	GAGTCGTACACCTTGCACTG
**CLDN5**	AGAGGCACCAGAACCAATTC	CATCCTACCAGACACAGCAC
**CLDN8**	AGCTGGATACAATTTGGGAGG	CCACTGAGGCATGATAGTCAC
**CLDN1**	GAATTCTATGACCCCTTGACCC	GATCTCTTCCTTTGCCTCTGTC
**ZO-1**	GATGAGCGGGCTACCTTA	CATGCGAGCGACCTGAAT
**OCLN**	GTTCTGCTTCATCGCTTCC	AGTCGGGTTCACTCCCATT
**IGFBP1**	CGCCATCAGCACCTATAGCA	TGTAGATTTCATCTCCTGCT
**IGFBP4**	TCATCCCCATTCCAAACTGTG	GCTTCACCCCTGTCTTCC

**IGFBP5**	AGGTGTGGCACTGAAAGTC	TGTGACCGCAAAGGATTCTAC
**IGF1R**	TTGTGTGTGTCCTGGATTTGGG	GCAGAAATGCGGAGTGGAAATG
**CREBH**	GGCCATTGACCTGGACATGT	TTCACAGTGAGGTTGAAGCGG
**IL-6**	GTCCTTAGCCACTCCTTCTG	TTCCATCCAGTTGCCTTCT
**IL-1β**	TGGGCTGGACTGTTTCTA	ATCAGAGGCAAGGAGGAA
**TNFα**	GTCTCATTCCTGCTTGTGGC	GCACTTGGTGGTTTGCTACG
**18s**	TAAGTCCCTGCCCTTTGTACACA	GATCCGAGGGCCTCACTAAAC
**Human**	Forward (5`-3`)	Reverse (5`-3`)
**CLDN1**	GAATTCTATGACCCCTTGACCC	TGGTGTTGGGTAAGAGGTTG
**CLDN2**	TCTTCCCTGTTCTCCCTGATAG	TCTTGACTTTGGGAGGTTGAC
**CLDN3**	CATCACGTCGCAGAACATCT	GAGTCGTACACCTTGCACTG
**CLDN5**	GTCTGATCCTGGCGTGC	AGTCGTACACTTTGCACTGC
**CLDN8**	ACCCATGCCTTAGAAATCGC	CATCCACAGTCCTTCCCAG
**ZO-1**	ACTCCACCGGAGTCTGCCAT	ATCCAGGAGCCCTGTGAAGC
**OCLN**	CAGGCAGCCTCGTTACAGCA	TCGCCGCCAGTTGTGTAGTC
**IGFBP1**	GAAGGAGCCCTGCCGAATAG	CCATTCCAAGGGTAGACGCA
**IGFBP4**	TCTGAGCCCTGGTGTTTC	GCTGGCACGTAGTACATGGT
**IGFBP5**	TGTGACCGCAAAGGATTCTAC	AGGTGTGGCACTGAAAGTC
**IGF1R**	TGTCCAGGCCAAAACAGGAT	CAACCCTCCCACGATCAACA
**CREBH**	CCTCTGTGACCATAGACCTGG	ACGGTGAGATTGCATCGTGG
**IL-6**	GTAGTGAGGAACAAGCCAGAG	GAACTCCTTAAAGCTGCGC
**IL-1β**	GCTCTCCACCTCCAGGGACA	AGGCCCAAGGCCACAGGTAT
**TNFα**	CCTTCCTGATCGTGGCAG	GCTTGAGGGTTTGCTACAAC

Table S2. Scoring system for disease activity index

Score	Body weight loss	Stool consistency	Bleeding
0	none	none	none
1	0-5%	---	trace
2	5-10%	loose stool	mild hemoccult
3	10-20%	---	obvious hemoccult
4	>20%	diarrhoea	gross bleeding
